# Negotiating Priorities on the Shopfloor: A Design Case Study of Maintainers’ Practices

**DOI:** 10.1007/s10606-022-09444-5

**Published:** 2022-10-15

**Authors:** Christoph Kotthaus, Nico Vitt, Max Krüger, Volkmar Pipek, Volker Wulf

**Affiliations:** University of Siegen, Kohlbettstr. 15, 57072 Siegen, Germany

**Keywords:** CSCW, Maintenance, Repair, Design case study, Qualitative research, Manufacturing

## Abstract

The coordination of maintenance work in manufacturing poses a crucial productivity factor in small and medium-sized companies (SMEs) but often seems to be rather neglected in practice as well as in much of the literature on maintenance. We shed light upon maintenance coordination work by presenting a design case study conducted in an SME over approximately two years. We took a participatory design-oriented approach, involving all roles on the shopfloor affected by maintenance work. In three major iterations during the pre-study, a release-ready prototype was developed and implemented by the users over the course of one year. The evaluation of the tool showed how a new and mostly unintended practice of information flow, error reporting, and prioritization emerged such that, for instance, foremen becoming a central node of communication, formal prioritization shifting away from higher management, and actual prioritization being done by maintainers. This paper contributes to the body of CSCW work on maintenance practice in SMEs by presenting detailed empirical findings on the coordination work of maintainers, as well as the evaluation of socio-technical interventions into maintenance practices.

## Introduction

The maintenance of production equipment is recognized as being of great importance in manufacturing small and medium-sized enterprises (SMEs) being highly significant for productivity and competitiveness (Maletič et al., 2014; Paz and Leigh, 1994). However, supporting the coordination work of maintenance remains rather neglected or, indeed, controversial in SMEs (Tan and Gable, 1998). Much of the literature treats maintenance work as a management problem, such that modelling workflow is presumed to determine maintenance outcomes (e.g. Paz and Leigh, 1994). Alternatively, maintenance work has been treated as a ‘knowledge management’ problem, such that propositional exchanges are seen as the main problem. Here, we treat maintenance as a coordinated activity, involving a number of different actors than maintainers themselves. The discrepancy between highly sophisticated maintenance models on the one hand and the actual practices to be found in SMEs on the other needs investigation in more detail (Ko et al., 2005), if the competitiveness of SMEs is to be supported in light of contemporary digitalization challenges (Ludwig et al., 2018).

This paper sheds light upon maintenance coordination work by presenting a detailed design case study (Wulf et al., 2011, 2015) conducted in an SME over approximately two years. The study was carried out as part of a larger project aiming to support SMEs with digitization issues. We were contacted by the specific SME at the heart of this study to address challenges related to their maintenance processes. It is crucial to point out that our aim and task was not to develop an innovative product or technology but to develop a *process innovation* supported by a *technical intervention*. The company already used some software in the company to, e.g., document the inventory of the maintenance department or the times that machines were checked. It was not clear, however, whether other commercial software would resolve the issues that the company experienced. Our aim, then, was to understand the practices of workers and maintainers in this process and design a support infrastructure for their work. Subsequently, as we recount below, it became clear that implementing a lightweight solution, mainly developed by us, was a preferred solution. We took a user-centered, participatory design-oriented approach, involving all roles on the shopfloor affected by maintenance work. First, a qualitative pre-study in form of interviews and observations was conducted. Based on these findings, an iterative and user-centric design process was conducted, drawing on co- and participatory design approaches (Kensing and Blomberg, 1998; Sanders and Stappers, 2008). After several design iterations including evaluation workshops, a release-ready prototype was developed as a progressive web app (PWA, see footnote in Sect. 4), drawing on an existing open-source system, that can be used on handheld devices (smartphones, tablets) and desktop computers. This prototype was then used by shopfloor staff for approximately one year for organizing maintenance-related tasks. During this period, two situational evaluations (Twidale et al., 1994) were conducted, focusing on the socio-technical effects of the new tool, and accompanying changes in the practice of maintenance. These qualitative evaluations were supported by a small statistical analysis of system usage.

The evaluation of the tool showed how a new and mostly unintended practice relating to information flow, error reporting, and prioritization emerged. Workers were, in the system intended to enter tickets directly as the need emerged, providing a fast flow of information. Foremen and higher management were to be made aware through the system. The emergent practices, however, showed a shift of the locus of communication. Only foremen were equipped with the necessary devices for entering tickets into or retrieval from the new system. In addition, maintainers refused to take on assignments other than through the new tool, forcing workers to report any maintenance issues to their foremen. Furthermore, where initial study showed a preference for prioritization to be shifted to higher production management levels. Subsequent evaluation showed that initial prioritization of tasks in the new system was rarely changed and the first assessments by those entering new tickets remained significant. Thus, formal prioritization shifted to lower instead of higher management levels.

This paper contributes to the body of CSCW work on maintenance practice in SMEs by presenting detailed empirical findings on the coordination work of maintainers. The study shows how the coordination of maintenance tasks is organized in an SME and suggests design implications for a lightweight, but mature tool design capable of supporting everyday maintenance coordination. This allowed for an extensive evaluation over the course of one year, allowing for better understanding of coordination work and its effects on maintenance coordination practice.

After providing an overview of the existing literature on maintenance, including literature from management and CSCW research, the background of the study as well as the methods are summarized. Following this, the empirical pre-study and the user-centered iterative design process are described, resulting in a concrete concept and implementation of a tool for supporting maintenance coordination. Afterwards, the results of the evaluation are presented, followed by a discussion and conclusion.

## Related work

Maintenance as an object of scholarly and practical interest has been studied from a variety of angles, by different disciplines and in different domains. The existing literature is accordingly vast and diverse. Despite divergent findings in such different domains as software maintenance and industrial maintenance, similarities can also be identified. Studies from the maintenance of software have proposed ‘corrective maintenance’—maintenance in relation to failure; ‘adaptive maintenance’—maintenance in the face of environmental changes, and ‘perfective maintenance’—performance enhancements, as a useful categorization for different forms of software maintenance (E. B. Swanson, 1976). Any kind of maintenance, however, is likely to be hugely disruptive, as systems need to be continuously up and running (see e.g. Swanson, 1976). We find this echoed in our own experiences, despite the fact that we are not dealing with software maintenance. Much of the maintenance work our study focusses on fits neatly within the category of ‘corrective maintenance’. the maintenance as well as the malfunctions themselves are—perhaps quite obviously—disruptive to production.

This is further complicated by the finding that—within the realm of software—maintainers and managers have often radically different attitudes towards maintenance (Tan and Gable, 1998), and considerable variations in knowledge regarding the kinds of problems and tasks maintainers faced. Work by Kemerer and Slaughter (1997) echoes these findings, pointing to a gap between technical and managerial knowledge, where ‘managers have very little knowledge about the types of maintenance work that are likely to occur’ (Kemerer and Slaughter, 1997). As a result, Ko et al. (2005) draw attention to the need for detailed studies on the different ways in which maintenance tasks are actually accomplished.

### Maintenance in industry and production

Most relevant to the context of this paper are studies from the domain of industry and production. In this domain, a different classification of maintenance activities has been proposed (Bateman, 1995; Paz and Leigh, 1994). These types of maintenance include reactive maintenance or failure-based maintenance (Gits, 1992), when machines are repaired upon breakdown, and preventive maintenance when machines are repaired periodically at scheduled times to prevent breakdown. A third type is predictive maintenance. Predictive maintenance relies on specific diagnostic techniques that signal imminent breakdown which can then be prevented through repair. (Swanson, 2001) has proposed aggressive maintenance, or total productive maintenance (TPM), as an alternative fourth form of maintenance, aided by global competition and technological advancements. Different kinds of maintenance (e.g. reactive vs. predictive) include different practical activities and embody different organizational priorities, as studies of maintenance show (e.g. Orr, 1996).

Studies in this domain have frequently been conducted from a managerialist, top-down perspective, treating maintenance as solely an issue for management. To be clear, we do not intend to suggest that all managers share this perspective, but that a specific strand of literature exhibits such a view. This literature reports that maintenance is frequently treated in organizations as a necessary evil (Cooke, 2000; Duffuaa and Andijani, 1999; Mobley, 2011). This is, for example, embodied in the concept of Total Cost of Ownership (TCO Analyst, 1997) when applied to maintenance. TCO is the attempt to calculate all costs of owning and running machinery or equipment, to arrive at ‘a holistic view of costs related to IT acquisition and usage at an enterprise-level’, as formulated by the Gartner Group (TCO Analyst, 1997). Such a holistic view would include the cost of materials, space, and other infrastructure, and also breakdown, maintenance, and repair (Castellani et al., 2005). This perspective then further removes maintenance from ‘primary’ productive processes, highlighting its costs and enabling, for example, outsourcing to providers that promise lower maintenance costs. Following, the concern of this literature frequently is the absence and development of best practice, such as the failure to introduce proper policy and procedure, as highlighted by Wireman (2004). They especially connect failures in production to management problems such as a lack of failure analysis, under-skilling, a lack of communication, poor use of materials, poor inventory control, and a poor assessment of maintenance costs as calculations frequently exclude the costs of machine downtime. Furthermore, addressing maintenance issues from a management perspective and developing managerial best practices requires accurate measurement systems (Raouf and Ben‐Daya, 1995), which are not always in place. While such management-oriented work certainly highlights challenges we find echoed in the specific context under investigation in this paper, it also exhibits critical shortcomings. Treating *management of maintenance* as the primary activity under investigation, much of this work focuses on the challenges of implementing appropriate management strategies such as implementing TPM (Cooke, 2000), overcoming challenges related to ‘culture’, where culture remains underspecified (Harrison and McKinnon, 1999) or trust, dependability (Avizienis, 2000) and reliability (Nakagawa, 2005). Little however is said about maintenance as a primary activity and thus, as Márquez (2007) points out, this literature might be focusing on doing ‘the wrong thing right’, as effects on actual maintenance remain understudied. As has been pointed out by Castellani et al. (2005), a purely cost-oriented approach such as embodied in, for example, TCO, is not able to capture the complexity of the connectedness of certain tasks and activities appropriately. Fucks and Dien (2013) have expressed a similar criticism with regards to the role of rules and procedures in the management of risk, such as the risks of machine breakdown. While procedures surely play a crucial role in managing risks and safety, they do not guarantee safety, nor do they prevent breakdown. On the other hand, there is also a risk of ‘over-proceduralization’, which limits peoples’ behavior to respond adequately to situations, for example those that are not specified by procedures. Our own study here further illustrates this by highlighting the interdependent and collaborative nature of maintenance work at an SME.

Digitalization associated with industry 4.0 or IIoT also has clear implications for maintenance. New technical developments have recently been extensively deployed, supported through the massive collection of data, and are assumed to support maintenance work effectively. A developing literature demonstrates this technological focus (see e.g. Brundage et al., 2018; Daily and Peterson, 2017; Jahnke, 2015; Lee et al., 2020; Sexton et al., 2019; Siltanen and Heinonen, 2020). Equally, management decisions continue to have a demonstrable impact on how maintenance is to be addressed. What is less clear is whether such developments have value for resource constrained SMEs. Digital processes, that is, need to be developed with respect to organizational processes which, in turn, need to be cost-effective. This gap, we suggest, highlights the need to investigate maintenance practices directly. With our study we respond to this by directly studying the practices of maintainers and maintenance within an SME and, building on these findings, investigating the possibilities for supporting maintenance practices with digital technologies from a user-perspective and directed towards a process innovation.

### Direct investigations of maintenance in CSCW

A classic study in this regard is Julian Orr’s study of the repair of photocopy machines (Orr, 1996). The key findings of Orr, previously neglected elsewhere, include that maintenance problems are heterogeneous, varied, and unpredictable, that expertise is distributed between maintainers and not all maintainers are equally skilled for the same task, that maintenance can be done for a variety of goals or with different levels of intensity, depending for example on available time and that maintenance is cooperative work. Building on this, several studies have continued to investigate the actual problem-solving processes of service technicians (e.g., Bobrow and Whalen, 2002; Yamauchi et al., 2003). These studies illustrate the complex knowledge practices that service technicians employ when fixing print- & copy-machines. Rather than following standard procedures, technicians employ a variety of techniques to understand and ‘probe’ the specific problem. They draw on a variety of information sources, most importantly the advice of their colleagues, to gather and combine the knowledge required to address the specific problem. The system at play in these specific studies facilitates this knowledge process, relying on and supporting the expert knowledge of the technicians, rather than prescribing specific procedures, as top-down approaches might suggest.

CSCW and the wider HCI community have extended investigations into maintenance practices, by trying to understand practices of maintenance better as well as supporting such practices through digital tools. Within HCI and Science-Technology-Society Studies (or Science and Technology Studies—STS) a specific strand of literature has investigated maintenance and repair practices in rather precarious circumstances, building on the idea of ‘broken world thinking’ (Jackson, 2014). Such studies have investigated for example technology repair contexts, especially of mobile phones, in Bangladesh (Jackson et al., 2014), Kenya (Wyche et al., 2015), Ghana (Burrell, 2012), Paraguay, and the USA (Rosner and Ames 2014) or the upkeep of local community network infrastructure in Cuba (Dye et al., 2019). Much of this work highlights the generative capacity of repair and maintenance work as an object of interest for HCI studies, contrasting it with the predominant focus on innovation (Russell and Vinsel, 2018).

Closer to the issues at hand, several studies within CSCW and wider HCI have investigated maintenance in industrial contexts. Such studies—somewhat unsurprisingly—frequently focus on the role of digital technologies in these settings and reported for example on the design and use of Augmented Reality systems for machine set-up (Hoffmann et al., 2019; Pinatti de Carvalho et al., 2018) and maintenance (Aromaa et al., 2016; Ferrise et al., 2013; Nee et al., 2012; Re et al., 2014; and to a certain extent Hannola et al., 2018) or of smartwatches and other wearables to support maintainers and maintenance (Lukowicz et al., 2007; Siegel and Bauer, 1997; Zheng et al., 2015a, b; Ziegler et al., 2015). Betz (2010) studied machine breakdown within an injection molding plant. Their study focuses on the management of knowledge necessary for maintenance and repair, such as the documentation and sharing of the history of breakdown for each individual machine, and access to additional internal and external expertise. Similarly, Hedjazi (2018) studied maintenance practices and the opportunity for CSCW systems to build collective competence with regards to maintenance tasks by involving internal and external expertise and by supporting various knowledge practices between involved actors. Lutters and Ackerman (2007) investigated repair practices in aircraft maintenance, although in their study maintenance merely served as a pretext to study the role of boundary objects within industrial organizations and their influence on organizational memory. Within the context of the advent of ‘Industry 4.0’ or the digital transformation of industry (Lewkowicz and Liron, 2019), predictive maintenance has gained some renewed attention (e.g. Wurhofer et al., 2018). With the increased availability of distributed sensor network and automated analysis of resulting data or machine learning algorithms (Silvestrin et al., 2019; Susto et al., 2015), cyber-physical systems can detect anomalies in the functioning of machines, signaling impending breakdown and can alert maintainers to prevent it (Börütecene and Löwgren, 2020). It needs to be said, however, that predictive maintenance does not rely solely on technological sensing capabilities. The trained senses of human maintainers are often able to similarly detect anomalies, such as ‘funny sounds’ in machines and react, as has been pointed out much earlier (Mobley, 2011). Ho et al. (2006) proposed a technical mechanism to schedule maintenance of railway systems, based on modelling e.g., stress, ageing and environmental effects on infrastructure components, an algorithm proposes ideal maintenance intervals. A study by Harding et al. (2019) investigates the relation between drainage maintenance practices on one hand and data work and IoT systems on the other, drawing on ‘classic’ CSCW concepts such as Awareness or Articulation Work. Their study also issues a small warning about automation in maintenance work, as it might lead to a loss of understanding and control amongst maintainers. A body of work has investigated trouble-shooting and problem-solving as a form of maintenance, especially remote troubleshooting (Crabtree et al., 2006; O’Neill et al., 2005a, b; Tolmie et al., 2004). Some of this work has explicitly focused on problem-solving as embedded in articulation work (Crabtree et al., 2006). While that study focusses on the complexity of articulating and understanding what the problem is, it highlights the profoundly coordinative nature of maintenance and repair work. This coordinative nature is thus further illustrated by our study at hand, albeit focusing on a different aspect: deciding what to do next.

Together, these studies clearly articulate a need for practice-oriented research, they highlight the collaborative nature of maintenance work and the promises of computer-mediated support. Nevertheless, the number of articles within the CSCW community dealing specifically with maintenance in industrial setting remains rather small. Our study, therefore, makes an important contribution to the understanding of maintenance practices and the role of digital support tools in industrial contexts of the twenty-first century.

As we will show, however, the specific challenges maintainers in our context face are related to coordination, negotiation, and scheduling—topics, on which the CSCW literature has much more to say. A standout study in this regard is early work by Button and Sharrock (1997) on the production of order in an industrial printing plant. The study details how (without the use of computing systems, it needs to be said) an order of production is created, with the help of three specific devices and the two imperatives that printing jobs should not take longer than ten days and machines should always be busy. They point out the difficulties associated with introducing a new system as it necessarily also imposes organizational changes on the context, by now commonplace understanding in CSCW. This is similar to the challenges that maintainers in our context face when deciding which repair job to do next, which represents the production of order in maintenance tasks. A study by Rouncefield et al. (1994) investigated routine work in a small office considering the implementation of digital systems into their workflow. Although the digital technologies small (or large) offices have at hand today are not comparable, the workers in the office were faced with constant interruption, similarly to the maintenance workers in the present study. However, while the office workers perceived the interruption as a distraction from their actual work, the interruptions the maintainers are presented with are very much part of their actual work (usually they are requests for maintenance work to be done), but the unordered and informal nature in which they are confronted with these requests makes prioritization and coordination of work difficult. The concept of ‘organizational acumen’ (Tolmie and Rouncefield, 2016) is also of relevance here, understood as the ability ‘to know how to appropriately arrange one’s actions and interactions’. In their work, Tolmie and Rouncefield (2016) illustrate the local, specific, situational kind of knowledge that members of an organization such as a small company need in order to prioritize tasks or create descriptions of organizational processes. These processes, as they show, are not systematic, rational reductions to what is found to be most efficient, but are contingent, depending on the specific situation in a specific organization and the specific members involved. Sala et al. (2019) described the highly individual process maintenance represents in a company and that an efficient support of maintenance through modern technology relies on the capability of collecting various data adjacent to the specific context. In our case, the design of a tool to assist maintenance processes can similarly be perceived as the attempt to create a description or even prescription of these processes, how to coordinate and how to prioritize tasks. Crucially, then, it needs to take into account the members’ knowledge and expertise in making situated decisions, acknowledging that processes cannot be prescribed, not even by e.g., line managers entering maintenance tasks into the system as orders for repair. What gets done exactly and when, is the result of a negotiation between different members that occurs in a specific moment, as we will show.

Building on this work, our own study presents a pragmatic real-world investigation of the actual challenges maintainers in an SME face as well as the consequences of the design of a CSCW system to intervene in maintenance practices. This pragmatic approach presents a contrast to top-down perspectives found in management-oriented studies outlined above. Our main contributions are thus an empirical account of maintenance practices, the outline of a CSCW system to improve prioritization of maintenance tasks and a report on the consequences of the introduction of this system. We do not provide new concepts or theories but measure our findings against existing concepts and literature. Our study is a contribution to the CSCW corpus on maintenance-related work and provides insights on the affordances of digital technologies to support maintenance practices for practitioners (who have requested the project which has led to the activities described here) and CSCW researchers and designers alike.

## Background

To foster projects concerning digital transformation in German companies, especially SMEs, multiple publicly funded initiatives try to bring the innovative start-up-scene, universities, and well-established companies together, to work on ideas, to find new business cases or just to shine a new light on stagnated processes and support companies with their digitization efforts. The specific project this paper discusses was part of our work in one of these funded projects, which supports the digitization of regional companies. Within this project, the research group offers several services to local companies, from informational events to series of hands-on workshops to small scale research and design projects, which are meant to explore and illustrate what digitization could mean for a company, which effects it could have and how a company could initiate digitization means for themselves. These projects take the form of rather small co-design projects, in which a specific challenge that the company faces, is identified and then prototypical technological applications that address this challenge are developed together. Due to the specific structure of the program and the requirements of the funder, no longer-term research projects are possible. Despite the time constraints this imposed on our work, the present study covers a pre-study, a co-design phase, the implementation, and evaluation of a fully functional prototype, which has been in use since July 2019.

After initially leading the project and being our contact in the company, the head of production handed the coordination tasks and project leadership over to another employee. This employee had the role of a digitalization manager: In the traditionally structured company, he implemented a specialist department for digitalization issues, decoupled from the IT-department. The aim was specifically to develop a digital strategy for the company rather than troubleshoot daily problems. As a former employee in the organization’s IT department, the digitalization manager knew the structure of the company very well and had a major influence on the plans for projects aimed at more digital production, management, and overall organization solutions. Through his endeavor the overall awareness of the potential of digitalization (what is called IIoT or industry 4.0) was to be raised throughout the whole company and our project was further pushed. For the company, the project had the spirit of something experimental, novel, and risky but also innovative. He was present from the beginning of the project and was deemed to be our formal contact for further coordination.

**Figure 1.**
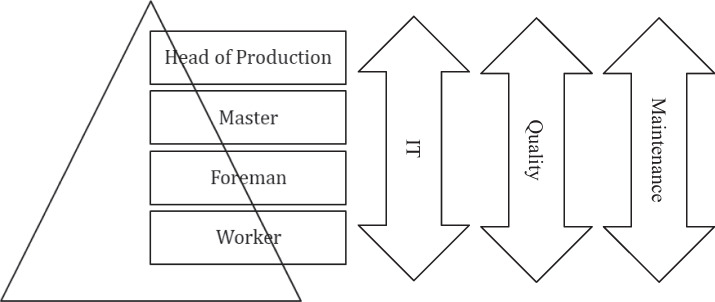
Hierarchy in the company for production processes. Maintenance as a secondary process is aligned vertically over all hierarchies and along all processes

Our study took place in a small and medium-sized enterprise in a rural area of western Germany in the state of North Rhine-Westphalia. Like many family-owned businesses in the region, the company has a long tradition, originating with building tools for the coal and steel industry in the nineteenth century to constructing tools for manufacturing halls and heavy-duty lifting today. The company still has a production site where production began 150 years ago. A second facility was built in a nearby industrial area. The main production processes were transferred to this new site, but the structures remained largely unaltered and were typical for this kind of SMEs: Organizational hierarchies follow a traditional structure of workers, foremen, master craftsmen and heads of production (see Figure 1). Work is organized into shifts, and each shift has a shift leader. Every shift has also a person who is the contact person and leader.

In one of our first meetings at the company, the head of production explained the scope of the project and discussions took place as to who should be involved. We argued that all hierarchical elements should be present in the project, as maintenance practice tend to implicate decisions made at all levels.

Furthermore, tasks are typically divided into primary and secondary tasks. Primary tasks are directly concerned with production. Secondary tasks are supportive tasks, not directly concerned with production. Maintenance is regarded as a secondary task. It therefore exists outside of the hierarchies of production and has no clear authority to issue instructions e.g., to workers on machines. During our study, three employees were working for the maintenance department: Two of them in full-time employment and one person only upon request in case of electrical issues, as only he had the necessary qualifications and certifications to handle electrical equipment.

As a manufacturing company, employees in the production processes work in shifts. During normal workload based on orderings the company would work in two shifts – one early shift from 6 am to 2 pm and one late shift from 2 to 10 pm. As only production line employees are working during the two shifts, secondary processes, such as maintenance, are only staffed in the early shift, between 8 am and 4 pm. In the late shift no maintainers are present, despite the fact that machine breakdowns might of course still occur. These usually need to be dealt with the following morning and are brought to maintainers’ attention by informal notes that workers or foreman place on their table or tell them about in the following shift. The workload of maintainers is usually determined by two types of work: (1) The work that has to be done on-demand as when there are breakdowns and (2) self-determined processes like scheduled maintenance or other projects that maintainers are working on.

## Data collection and method

During one of our events the head of production explained current pain-points present in his work concerning the maintenance department and the insufficient flow of information between the different stakeholders in the process. In order to address this issue and considering the time constraints imposed on us by the overall project structure, we adopted a Design Case Study (DCS) approach (Wulf et al., 2011, 2015). Grounded within a *practice-oriented* approach to HCI (see also for example Kuutti and Bannon, 2014). DCS is a user-centered framework for the design of socio-technical systems. It covers an ethnographic investigation into practices as the foundation for design work as well as investigation into the appropriation of the designed technological applications, using qualitative methods such as interviews and observations. The DCS framework is organized along three different phases: 1) a pre-study that investigates the specific use-context and practice in question, challenges practitioners face and specific design needs. 2) a design phase in which technological applications are designed, ideally in close collaboration with practitioners based on the insights from the pre-study and 3) an appropriation phase during which the designed artefacts are introduced into the practice context and the evolving use closely observed in order to understand its effects and any resulting changes in the specific practice. In this paper, we use both the term ‘practice’ as well as ‘process’. Process refers to an idealized and simplified description of a part of a practice, which refers to the situated and varied actions, artefacts, context, motivations and relations involved in maintenance practice (Kuutti and Bannon, 2014). This type of study can be considered ‘in the wild’ design research, taking place outside of the laboratory (Chamberlain and Crabtree, 2020). Such studies are in line with much of the work within CSCW and akin to other disciplines’ common approaches to qualitative field work. The entire study presented in this paper took place over the course of 12 months. In accordance with this approach and its different phases, the study began with an ethnographic inquiry into the maintenance practices of the company. Over the course of 2 weeks, we interviewed 11 members of the company, including maintainers, production workers, foremen^1^ and managers, in order to gain a situated understanding of maintenance practices as well as related production practices. In unstructured interviews we inquired about their specific practices, any tools—digital or otherwise—used in their work, communication with their colleagues, handling malfunctions in day-to-day work, routines built around it, as well as any difficulties they experienced. Please refer to Table 1 for a full list of the participants and in which steps of the project these attended.^2^ All employees we interviewed and who participated during the workshops have a notable amount of experience from either being trained in the company itself or coming from other companies and already having completed a full job training elsewhere. All in all, it can be said that only experienced employees participated in the study, who are involved in the practice we wanted to study.

**Table 1.** Participants and their roles in the project. Due to the long duration of the project participants not always participated for every occasion. Column 3 provides an overview on who we had contact to.

Participant	Position	Occasion in project
P01	Master	1^st^ round of interviews, Oct. 2018 1^st^ workshop, Dec. 2018
P02	Head of production	1^st^ round of interviews, Oct. 2018 1^st^ workshop, Dec. 2018 2^nd^ workshop, Apr. 2019 3^rd^ workshop, May 2019 Shop-floor meeting, Sep. 2019 1^st^ evaluation, Oct. 2019
P03	Foreman	1^st^ round of interviews, Oct. 2018 1^st^ workshop, Dec. 2018
P04	Foreman	2^nd^ round of interviews, Oct. 2018 1^st^ workshop, Dec. 2018
P05	Head of production	2^nd^ round of interviews, Oct. 2018 1^st^ workshop, Dec. 2018 Shop-floor meeting, Sep. 2019
P06	Maintenance	2^nd^ round of interviews, Oct. 2018 1^st^ workshop, Dec. 2018 Shop-floor meeting, Sep. 2019 Error-fixing, Oct. 2019 1^st^ evaluation, Oct. 2019
P07	Maintenance	2^nd^ round of interviews, Oct. 2018 1^st^ workshop, Dec. 2018 Error-fixing, Oct. 2019 1^st^ evaluation, Oct. 2019 2^nd^ evaluation, Sep. 2020
P08	Worker	3^rd^ round of interviews, Oct. 2018
P09	Worker	3^rd^ round of interviews, Oct. 2018 1^st^ evaluation, Oct. 2019
P10	Worker	3^rd^ round of interviews, Oct. 2018
P11	Foreman	3^rd^ round of interviews, Oct. 2018 1^st^ workshop, Dec. 2018 1^st^ evaluation, Oct. 2019 2^nd^ evaluation Sep. 2020
P12	Work council	1^st^ workshop, Dec 2018
P13	Project coordinator from the company (CDO)	1^st^ workshop, Dec. 2018 2^nd^ workshop, Apr. 2019 3^rd^ workshop, May 2019 1^st^ evaluation, Oct. 2019 2^nd^ evaluation, Sep. 2020

In agreement with the company management, we focused our investigation not on the entire company but on one specific production line, which allowed us to gain a detailed understanding of the existing practices. Additionally, we participated several times in various regular meetings that were held for coordination purposes. Our observations from the participation in these meetings were documented in field notes.

The interviews were transcribed. The transcripts and the field notes were subsequently inductively coded, following a Thematic Analysis (Braun and Clarke, 2006) approach, to identify the main issues within the maintenance practice in this specific production line. Based on this analysis we outlined several implications for the design of technological interventions. A detailed version of the analysis is presented in chapter 5.

After the analysis was concluded and main issues were identified, we conducted a workshop with almost all members of the production line, excluding a small number of workers that had to keep the production line going. One representative of the work council participated in this workshop as well. In this workshop, we presented the major findings of our analysis and briefly outlined design possibilities, based on the implications previously identified. The presented design possibilities embodied different approaches to the support of the maintenance/error-reporting process within the production line and were meant to inspire the workshop participants and gain a common understanding of the opportunities. We discussed the findings as well as the design opportunities. The workshop then served to clarify our understanding and create a foundation for subsequent design efforts by collectively developing and agreeing on several design requirements.

Based on the outcomes of this workshop we developed the first prototype as a Progressive Web App based on the Angular framework^3^ and an existing open-source platform called OTRS.^4^ This prototype was subsequently presented to most of the participants who took part in the interview study and the workshop. From this moment on it was iteratively improved over several months, based on observing usage data remotely as well as observations and further interviews conducted within the company. The following table shows which data was collected on which time and during which occasion.

The second and third workshop were held together with the head of production and the project coordinator. They were interested in how far we had come with the development and wanted to test the app on their devices. The second and third workshop therefore took place at the university (the second one) and again at the site of the company (the third one). In both workshops further development aspects were negotiated.

As it emerged that the daily shop-floor-meetings were a crucial element of the coordination of maintenance, we wanted to participate in one of these and therefore observed the morning routine in September 2019. We had no active role and were not allowed to record anything because critical internal issues and decisions were discussed. One member of the maintenance department participated in this meeting as well as both representatives from head of production. Their participation was an important influence on our design regarding the filter mechanism to support quick interactions which we then later implemented (explanation of functionality in chapter 7.4).

**Figure 2.**
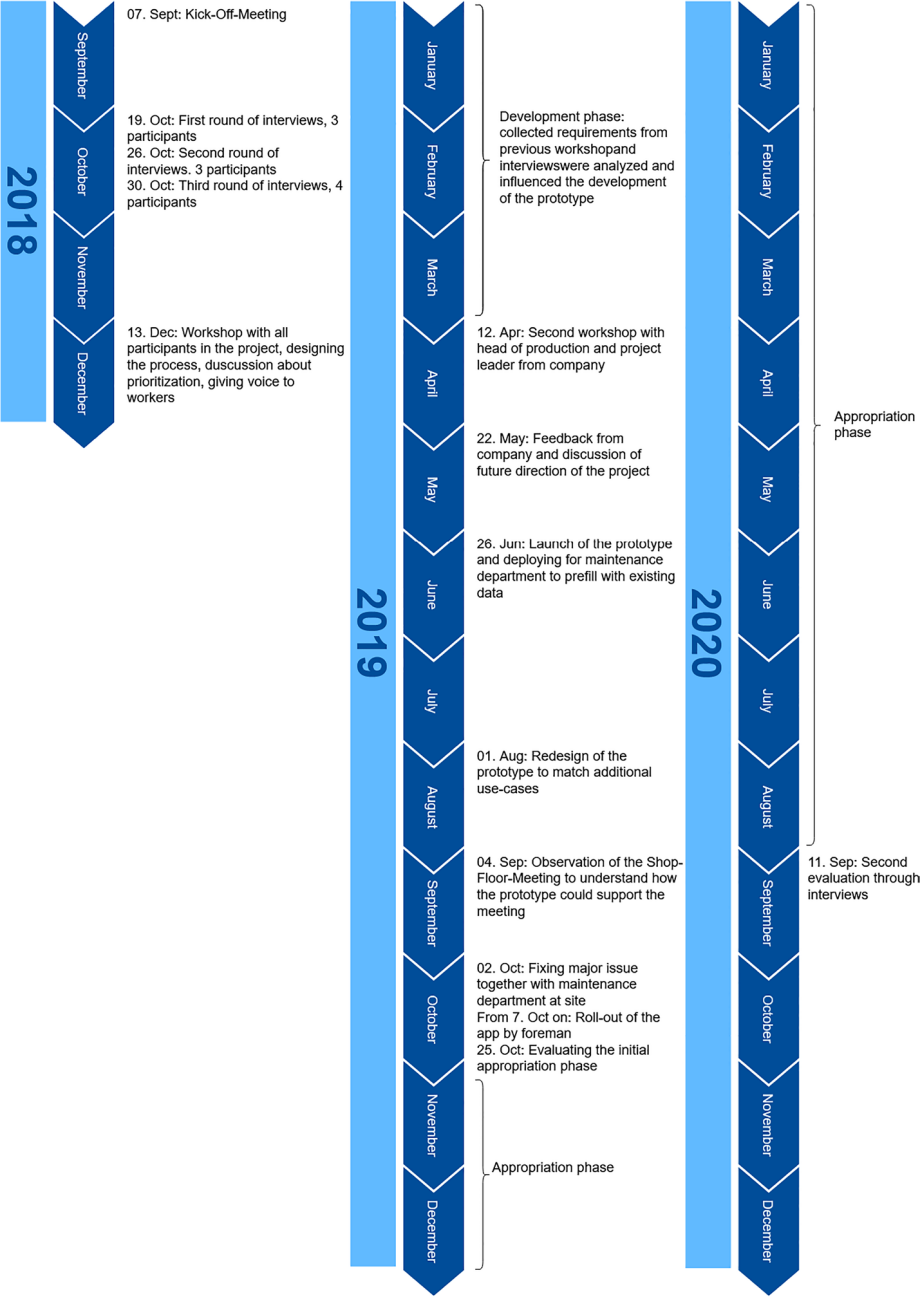
Project timeline with phases and major milestones

Due to an issue that emerged at the beginning of October 2019, we were forced to visit the maintenance department as we could not replicate the issue in our development environment. In this short meeting, we participated with both maintainers. The detailed timeline can be found in Figure 2.

In total we interacted with 13 employees of the company, holding various positions, from worker to manager. Participants were selected based on suggestions from management which initiated the project and were our initial contact point, as well as according to the availability and interest of the employees and our wishes to understand the perspectives of members of specific roles within the production line. For the purpose of this paper, all data was anonymized and subsequently shared with a limited number of colleagues. Drawing on Braun and Clarke’s Thematic Analysis (Braun and Clarke, 2006), the data was then coded by the authors. Codes were shared and discussed, a process which ultimately led to the narrative presented here.

## Maintenance practice

The site of the company where our study was conducted, incorporates a dedicated three-person department for maintaining machinery and equipment to support production and reduce downtimes of physical resources. This encompasses all mechanical and electrical issues not only regarding production assets, but also the periphery such as hall lightning or repairing roller shutters or fences. The maintenance department was implemented only approximately 5 years before this study, with the main objective of preventive maintenance, as we learned from the maintainers. As the production process requires similar skills to those of the maintainers, electricians or mechanics are partly able to solve issues themselves and are sometimes even asked for assistance in urgent situations. The production site, in general, is approximately 170 m long and 90 m wide, resulting in a rather distributed allocation of tasks. Due to the maintainers’ expertise, their job also includes the building of new prototypes or other equipment and facilities. Therefore, their department is equipped with relevant tools and a larger area within the production hall, located in its center. The basic elements of the maintainers’ work are machine errors (e.g., spontaneous malfunctions), planned tasks (preventive maintenance), projects (e.g. building prototypes), as well as an increasing demand for documentation.

The maintainers are variously skilled to cover the whole spectrum of requirements. The team consists of one electrician who does specialist work but also covers other assignments, one mechanic, who also leads this department, responsible for all other tasks, and one apprentice with limited permissions due to a limited skillset.

In the following, the maintainers’ work and coordination with connected departments is presented according to themes that emerged from the analysis of the empirical material.

### Currently used tools and their insufficiencies

The typical main task of this company’s maintainers is to regularly repair and overhaul production equipment (preventive maintenance) or to react to incidents e.g., machine breakdowns (reactive maintenance). To support this, they are provided with a simple portable telephone and specific maintenance software where they enter all important equipment details and scheduled intervals for regular preventive maintenance tasks. The maintainers also make use of its functionality to add handbooks and other documentation to each asset and assign tasks to certain users. The software is installed on a single desktop computer in their office, located in the maintenance department, and is only used by the three maintainers. It does not provide a mobile version to support the distributed nature of the maintainers’ tasks. The software version has reached its end of life due to bankruptcy of the vendor, which means that neither updates nor service support are provided anymore. The company decided to continue using this tool, as they are used to it and migrating data into a new solution seems too much of an effort. Another important technology used by the company is a standard ERP system, also containing some stationary terminals throughout the production site for production order confirmation. This software also provides functionality to enter breakdowns or other minor issues, mainly for the purpose of indicating deviations from estimated production times. However, this feature is rarely used by workers and there is little emphasis on enforcing this process. As a result, these messages are not used by maintenance and the possibility of recording issues in the software was discarded by production management in the past.

Coordination with other departments is rather unstructured and the use of tools is fragmented. This becomes even more problematic as maintainers are only available in the morning shift. In case of an incident in manufacturing in the morning shift, there is no formal procedure for reporting errors. As a result, workers, shift leaders or foremen tend to contact maintainers directly either via telephone or in-person by just coming by the department or whenever they see maintainers walking through the site, which often serves as a kind of memory trigger, reminding them of maintenance-related issues they have. There is basically no documentation of these incidents and maintainers try to remember and schedule these issues.

In case of malfunctions during the late shift, the available personnel try to fix the problem themselves or write an informal paper note to leave at the foremens’ or maintainers’ desk. Also, planned maintenance only rarely takes place in the late shift and is scheduled mostly for the morning shift. Maintainers do not follow the same shift schedule than the workers of the morning shift but start a bit later in the morning to stay for approximately one hour into the late shift to participate in a shift handover by the foremen and advise them in case of any occurrences.

**Figure 3.**
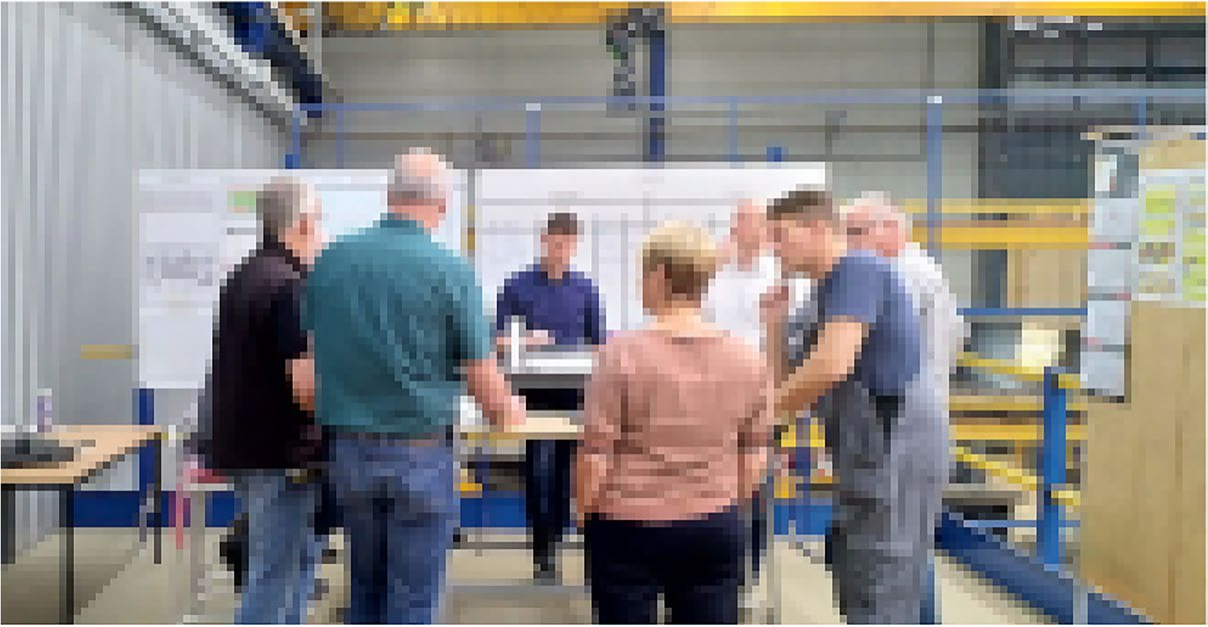
Shop-floor meeting with head of production, masters, foremen and other departments (pixelated for anonymity reasons)

In contrast to this rather unstructured practice, several coordination meetings have been established, called shop-floor meetings (see Figure 3). Every morning, foremen meet with their employees in their departments, checking whether all expected people are present, the status of production orders as well as the status of the machinery and equipment. Additionally, they check whether the late shift left any notes in their office on the keyboard. After this, all foremen meet with the masters for a separate shop-floor meeting in a separate area, discussing production orders, important incidents, or personnel.

**Figure 4.**
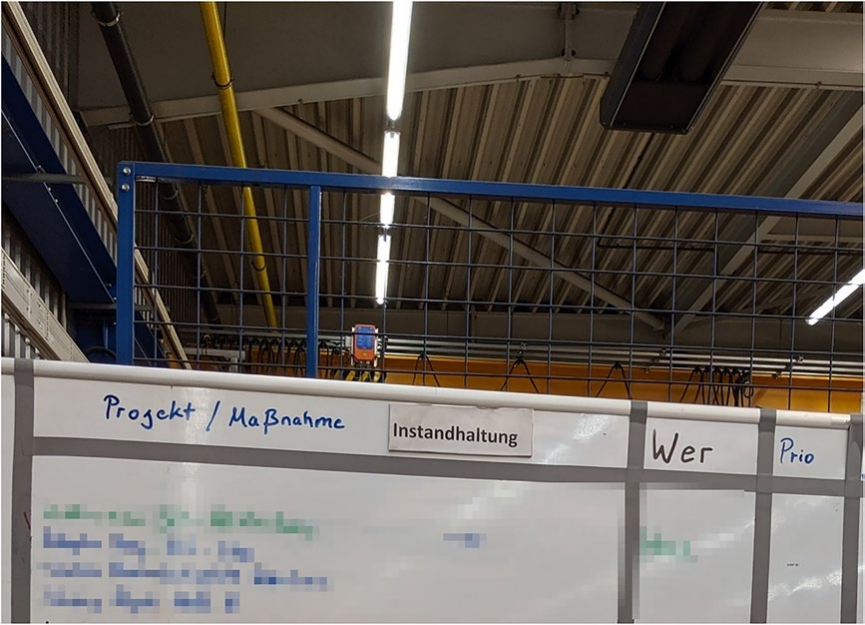
Whiteboard containing projects for maintenance (pixelated for anonymity reasons)

**Figure 5.**
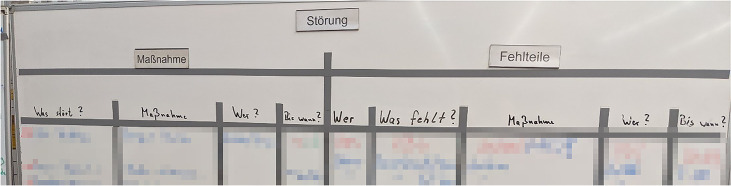
Whiteboard listing incidents and spare parts (pixelated for anonymity reasons)

The area is located in another production department on top of an office, appearing like a large balcony, and is also used for lunch breaks. For supporting the meetings, it is equipped with a D-shaped high table, one bulletin board showing statistics about production and quality management, and two whiteboards. One whiteboard gives an overview over projects (Figure 4) and the other for important incidents (Figure 5), both regarding maintenance. Both whiteboards are structured with duct tape and the entries are done with water-soluble markers.

Directly after the aforementioned meeting, the head of production, maintainers and members of the purchasing and quality management departments join the group for the final shop-floor meeting. This meeting is strictly limited to 15 min, leaving only a little time for each of the ca. 10 participants to report any issues. Thus, only the most important issues are mentioned by maintenance, and only these entries on the whiteboard are usually updated. The order of the issues on the whiteboard is never changed, and new issues are added wherever there is space left on the board. Due dates are assigned to issues and priorities set for projects to provide an order. However, this way of writing the issues down makes identifying priorities difficult.

According to the maintainers, the number of tasks they have to take into account is a lot higher than the number on the whiteboard, but the limited space, the cumbersome nature of the writing process and the remote location of the board neither allows for a comprehensive list nor a practical use in daily work. It is solely used for raising awareness in the daily shopfloor meetings and these issues are noted separately on paper by maintainers during the meeting. Although these meetings are well established and valued by the staff, they only allow for a limited understanding of the total number of maintainers’ tasks by production departments. Due to these shortcomings, the participants expressed a wish for efficient support of a new tool to be able to report and reorder issues also in the shopfloor meetings.

Fragmentation of maintenance and repair also exists in terms of available staff. Typically, maintenance is responsible for all such tasks. However, in the past, before the maintenance department was established, maintenance was organized in a decentralized fashion. There are electricians and locksmiths working in production, capable of carrying out some kinds of repairs. Before there was a dedicated maintenance department, these colleagues were asked for help in case of breakdowns. In the late shift or even in the morning shift, these colleagues are also sometimes asked for help informally, but also by the maintainers themselves in urgent cases. Thus, there is a legacy and overlap of competencies which provides a resource for informal workarounds. Qualified workers such as electricians are therefore part of other departments, working in shifts alongside the production chain. As there might be a shortage of these qualified workers, they are frequently asked to help out in the maintenance department on tasks only they are allowed to do.

‘For a while, we had a colleague from the electrical department, who usually stood at the back of the saw, for example, working in shifts. And you could just go and say here, the cable is broken, can you fix it. It was as simple as that. He was an electrician and was allowed to do that.’

P06 and P07 - Maintenance

We still have an electrical department; we still have ten electricians. If [a machine] doesn’t work at all or something like that, then I’ll contact the master craftsman in the electrical department. Yes, [P06] calls him and says, “Do you have the possibility [to come here] because I can’t get away from here and someone should have a look [at the broken machine].”‘

P06 and P07 - Maintenance

### Inconsistent flow of information

In the event of equipment errors, no formal procedure exists. The flow of information, hence, is up to individual and situated decision making, ranging from direct and detailed reporting to non-reporting.

Foremen and other managing staff members have a certain idea of how **reporting along the hierarchy** is to be done. Workers should report to their direct superiors, foremen or shift leaders, who then decide who to contact next. This would be either maintenance directly, and in cases with a larger impact on production flow also superintendents, who then decide whether to inform the head of production. The current practice, however, is that workers mostly contact maintenance directly in case of errors they either cannot fix immediately or do not want to fix themselves. From the maintainers’ perspective, the flow of information seems to be to some extent random. In cases that would take longer than approximately 15 min, the direct superiors are informed as well. Some workers, however, do inform their direct superiors directly, sticking to the unwritten, but expected way of communicating along the hierarchy, or even report to superintendents, when foremen are not available. Due to the size of the production site, it is not always possible to find foremen or shift leaders immediately. Also, only some workers have phones at their workplace to call them instead. When workers inform superintendents directly, the former will also tell maintenance to take a look at the problem. Foremen, however, will not be informed separately in most cases. Most foremen are not aware of this informal flow, assuming that almost all occurrences in their field of responsibility are being reported to them.

The subjective assessment of foremen is that they get to know all incidents in their area of responsibility and that all workers are aware of the expected flow of information and will mostly stick to it. The interview study, however, showed that **numerous incidents are not reported to them,** not even after the repair was done.

‘Yeah, so, they always let me know. Only sometimes they request a repair via me. Sometimes they make requests themselves and then just tell me about it. So, I know all about it.’

P04 – Foreman

Maintainers, however, do not feel responsible for informing foremen or other managerial instances, implicitly expecting workers to do so later.

‘Yes, well, the foreman, as I said, is then informed, just like the head of department when really nothing works anymore. […] But if [the workers] say that they can continue working like this, everything is okay, and we take of the problem eventually, then the foreman is not usually informed. At least not specifically from us.’

P06 and P07 – Maintenance

Although errors or even breakdowns seem to be minor to the workers at the beginning, leading them to contact maintenance directly, in some cases repair takes much longer, leading to longer idle times for the workers. Foremen need to know about such idle times because many workers are welders and could be reassigned to other workplaces instead. Also, they need to report the status of their field of responsibilities in the shop-floor meetings and estimate capacities for production orders as well as reporting issues to be noted and prioritized on the maintainers’ whiteboard (see Figure 5).

‘So, the wish is in any case that almost all breakdowns that affect the locksmiths, end up with us, because those cases usually include production downtime. Sometimes it happens that you are in a meeting early and say that this and that has not been accomplished. And then usually you have to go off and ask yourself, what happened? Why did this happen? How was it solved? So, it would be nice if the information came to us directly.’

P03 – Foreman

**Figure 6.**
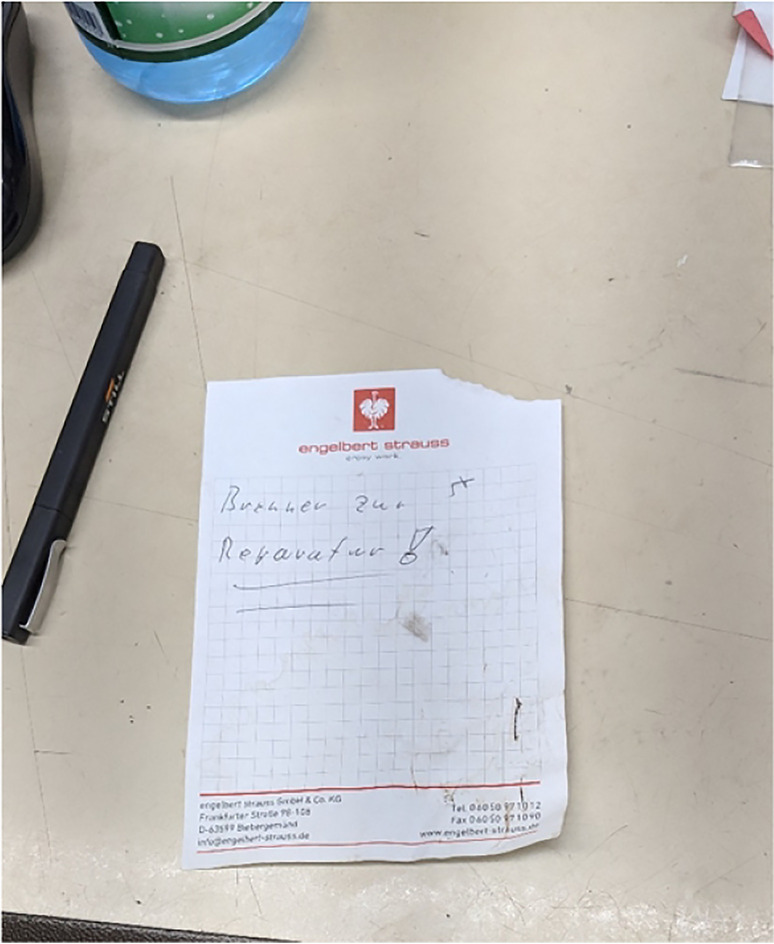
Paper note “[welding] torch for repair!”

Depending on the availability of maintenance personnel and individual preference, different **means of communication** are selected. During the morning shift, maintainers are contacted mostly by portable phone. Employees who are further away from the maintenance area choose this way of communication, to save the time and effort of going there. Due to the distributed nature of maintainers’ tasks, the chances of meeting them in their office anyway, so other employees also choose to call first. However, colleagues do not only call for requesting assistance, but also to request status updates about current issues. Workers and foremen who are closer to the maintenance area come by personally to report issues. Maintainers state that they tend to help directly in such cases, following them to the respective workplace to take a look and fix the problem immediately, if possible. Maintainers also state that there is a constant balancing act between satisfying these colleagues and maintaining the overall priority as discussed in the shopfloor meetings. Similarly, employees approach maintainers when they pass by their workplaces to report mostly minor issues or request status updates. During the late shift, where no maintainers are available and shift leaders take over the role of foremen, shift leaders or workers try to fix errors by themselves or leave informal paper notes either on the foremen’s or maintainers’ desk (see Figure 6), where they will be seen at the beginning of the morning shift. This procedure is well established amongst shopfloor personnel as a means for shift handover. The least explicit form of communication occurs when smaller devices break in the late shift. In such cases workers or shift leaders often place the broken device on the maintainers’ desk, sometimes even without a paper note, causing unnecessary troubleshooting efforts to maintainers, also by trying to find the respective colleague for consultation about the broken device.

‘Or the machines are simply placed here on the table, if they break during the late shift. We get there in the morning, there are three grinding machines lying here. Angle grinders. And then we don’t know what is wrong with them. Maybe it’s just a cable that’s broken? [P07] then has to test for every possible error.’

P06 and P07 – Maintenance

Especially during the late shift, when neither foremen nor maintainers are available, they are being called via smartphone or texted via **WhatsApp** in very urgent cases. Both channels are informally used. The phone numbers are not known to everyone, but only to trusted colleagues. In such a case, maintainers first try to solve the problem by guiding the caller through some steps where the latter describes the situation as good as possible. This is then often supplemented by sending photos via WhatsApp to help clarify the situation and to guide the caller through certain measures. If the error cannot be fixed or worked around this way and cannot wait until the next shift, most foremen or maintainers stated that they will come back to work. In our interviews the maintainers associated this with a sense of duty, often rooted in their long affiliation to the company. Maintainers are also interested in only work during their current morning shift, instead of working both shifts, and this is seen as a means to maintain this state. Also, the head of production wants to evaluate staffing the late shift regularly with maintainers based on the severity and frequency of issues in the late shift, mainly to not create unnecessary personnel costs.

In their effort to help themselves, especially during the late shift, employees sometimes take tools from other unmanned workstations in urgent cases, because spare tools are locked in a cabinet in the maintenance area. This causes inconsistent inventory and insufficiently equipped workstations, whose workers will then contact maintenance for assistance.

Workers or even foremen and shift leaders do not report errors at all if those can be fixed by themselves. This also includes cancelling error messages of machines, which can lead to more severe problems in the future. For example, one machine indicates a low level of lubricating oil 48 h before an emergency stop. This warning can be cancelled by anyone, so the machine will continue working. Mostly, this is not reported, and it is forgotten until an emergency stop is triggered by the machine, which is highly problematic in the late shift because nobody else, but maintainers are qualified to fix this.

All interviewees stated that they desire a consistent flow of information along pre-defined lines on the one hand, but also flexible and informal ways of communication on the other hand. Most of the interviewees are aware of the difficulty of prioritization and point out this as one of the most important organizational challenges regarding a new process.

### Assessing error reports

Due to the **unplannable occurrence of equipment failures**, the non-repetitive character of maintenance tasks (see also Paz and Leigh 1994) as well as the difficulty of diagnostics, it is sometimes neither entirely clear how severe an error might be nor how it can be fixed.

As our data shows, and as we have begun to outline already in the previous section, there is an ambivalent perception of responsibility for repair and manner of communication. This has severe consequences for maintainers when **assessing the correctness of error reports**.

The accuracy of these reports is an important factor, as reassurance by maintainers is time-consuming. To save time, they try to get a clear picture of the situation by asking the caller to describe the current situation and based on this, bring the right equipment. However, there remains a discrepancy between these accounts and the actual situation. According to the maintainers, these error reports are never adequate, but workers who mostly know their equipment very well tend to provide better descriptions than their superiors. Apart from oral descriptions, there are no other means in place for improving this first assessment such as sending pictures, videos or even machine logs.

‘Someone says: ‘you have to come here’. And someone else describes the error, but maybe the description is wrong, maybe not. You usually do not know that. You always have to weigh it up yourself, because you can quickly be led in the wrong direction.’

P06 and P07 – Maintenance

‘The shift foreman is somewhere in the [production line where pipes are manufactured] and the employee is an operator at the painting plant. Often the employee can describe the error better than the foreman. ‘

P06 and P07 – Maintenance

Furthermore, the unclear procedure or reporting errors also has consequences for maintainers’ ability to appropriately address errors, as it influences their capacity to detect root causes or causes problems when they are called in after the error has escalated.

Most foremen’s attitude is to first try to solve minor issues by themselves before contacting maintenance. This ought to save time for equipment recovery and spare maintainers from unnecessary tasks. Mostly, such issues are not reported, unless foremen and workers fail to solve the issue by themselves. In the latter case, maintenance is contacted to get help. Maintainers, however, see this procedure rather critical, because initial attempts to solve the issue could exacerbate the initial problem, or the root cause cannot be reproduced anymore. Also, at one of the bottleneck machines, some errors, such as warnings displayed by the machine, can be cancelled by the workers or foremen. Maintainers are then only contacted when the problem cannot be bypassed anymore. Identifying the root cause this way is exceedingly difficult as there is no log in the machine control to lookup previous messages.

‘And sometimes it is the machine itself, as was the case with printing. He [the worker] could have simply ignored the malfunction, switched it on again and not inform us. And that perhaps five times a day. He would have continued to work anyway, and we would not even know about it. So, we are now glad that he told us about it. Now we know that there could be a problem somewhere in the near future. Maybe it’s the compressor, maybe it’s something else. Because we already know that there is a problem. This happens very often, for example in the paint shop. Malfunctions occur there, they are pushed away, ignored, and the system continues to run. Sometimes the fault occurs up to five or ten times a day and we don’t know about it. That also happens. And that makes it harder to know what the problem is when we are called to fix something.’

P06 and P07 – Maintenance

Cancelling error messages also happens at other machines, but less often. Maintenance reacted to such a behavior by taking preventive action such as changing filters regularly to prevent more severe issues.

‘So, if the filters are now blocked or closed, or when they let in air. Then error messages come up briefly, you press away and then the machine runs for a while. At some point, it is on such a level that the error messages just don’t come back. That already happened in the past. Nowadays, we change the filters so regularly that this actually doesn’t happen anymore. But it did when a filter was full. Then at some point, the air looks for another way to get out. And when it has found its way, the pressure is right again. And then the error notification no longer shows up [although the error still exists].’

P06 and P07 – Maintenance

This ambivalence regarding problem-solving is also reinforced by the lack of maintainers’ presence during the late shift, leaving no other option for those present but to help themselves, which they then also tend to do in the morning shift, when maintenance cannot respond quickly enough. In very urgent cases, however, foremen or maintainers are contacted in their leisure time by the shift leaders. Either the case can be solved or worked around remotely, or maintainers come back to work to fix the problem. One foreman stated that he is also contacted by shift leaders in the afternoon or evening in minor cases and interprets this as a desire to show a sense of duty or to hand over responsibility for emergency actions.

‘No, both are usually done then as well. Only then they write me, so that I already know that evening. I don’t know why, because it doesn’t help much. But I do know already... maybe they feel better then.’

P04 – Foreman

In contrast to this rather proactive approach to problem-solving, some workers inform maintenance directly, often without informing their foremen. In turn, foremen state that some workers overreact, and indicating that some workers take more time than necessary or to act.

‘Yes, he does not bite down on a problem, but makes something big out of a small thing then spends a lot of time with the locksmiths. So, the problem can perhaps be solved in five minutes, it takes an hour, maybe.’

P03 – Foreman

Foremen and maintainers also state that they appreciate the workers own initiative for minor issues, but at the same criticize unwanted measures or the missing information from their superiors.

‘On the one hand, one is glad if the person can solve certain things independently without having to approach the foreman every time.’

P03 – Foreman

The general procedure by maintainers to first try to solve issues themselves is sometimes seen critically by foremen. In difficult cases, it can take days of error analysis or repair attempts until the decision is made to call for external help such as the equipment manufacturer or other service partners. This also indicates the foremen’s general attitude to keep production resources running and take things into their own hands rather than waiting for professional help, which often cannot react immediately, especially not in the late shift. In the late shift, foremen and. shift leaders try to get help informally from available workers of other departments, as already stated above. Similarly, foremen or workers take equipment from other workplaces, for example when a hand welder breaks. Because of undocumented exchange of such equipment from the storage located at the maintenance department, it was decided in the past to lock them away, leading to this kind of borrowing equipment, which is considered even more problematic, because it is mostly undocumented, too.

‘No. So as I said the angle grinder should not necessarily be a problem, in a midday shift for example. Even if they can’t get to the locksmiths in the back, because the spare tools are locked away. But even if you can’t reach them, you go to the workplace where no colleague is working in that moment and borrow a grinder.’

P03 – Foreman

### Forgetting error reports

The practice of reporting and handling issues as described above has another effect. Maintainers often forget issues that were reported by their colleagues, mostly on the way to some other section of the production area. Also, feedback to workers or foremen about the current state and estimated time of completion only rarely takes place by maintainers. This is not so much the case for very important issues, which typically are handled immediately, but is a problem for less important issues. Due to the lack of a consistent process and tools for noting down such issues, they can easily be forgotten. In some cases, this can even happen a few times for the same issues, frustrating colleagues, who then either do not repeat their request anymore or forward the issue to their supervisors, foremen or superintendents. In the former case, this can lead to the escalation of minor flaws to more severe issues, seriously affecting the flow of production.

‘It could be that you are told something on the side and then you forget it, as we just said. And then maybe we are informed about the same error twice. And sometimes the topic then goes to the foreman or superintendent or however and they learn that for six weeks the plant does not run like it’s supposed to, but we were not really aware of that. Because maybe we were told something once or twice but forgot, perhaps the coworker is frustrated then and does not tell us again. At some point, he has a bad day or something and then goes to the master.’ P06 and P07 – Maintenance.

‘Calling out to us [on the shopfloor] is one of those things that really gets lost. This is not a bad intention of us, but that is sometimes assumed. They’ve already told us about an error three times, but you suppress it a bit because it’s a completely different topic in that moment, and then at some point, you hear it from your superiors. The accusation that we don’t take care of things. Then we say, ‘why didn’t you write it down there?’’

P06 and P07 – Maintenance

This situation is also frustrating for the maintainers because forgetting requests is always unintentional and they want to maintain a good relationship with their colleagues. Maintainers also complain about the many disturbances during their shift e.g., many phone calls or informal personal requests on their workplace or on their way. This and the increasing demand for documentation regarding repairs leads to a feeling of being torn out of work and spending too little time for their perceived primary activity, preventive, and reactive maintenance.

In the past, some measures to report issues more systematically failed. At some important machines, paper-based structured lists were installed for entering all issues. However, after a few weeks, these lists were not used anymore and at the time of the interview, the list was not even mounted at the machine anymore. An alternative to this is to report on the whiteboard at the shop-floor meeting area, which is only rarely being done. Due to their perceived position in the hierarchy as colleagues instead of supervisors, they do not emphasize the adherence of these procedures.

‘Yes, that’s always a bit of a crossroads. No, we don’t want to be the ones who tell the employees what to do. Otherwise, we just make ourselves unpopular somewhere, and that’s not our thing. For us, it is only important that we have the knowledge.’

P06 and P07 – Maintenance

One strategy of the maintainers to avoid this is, as already described above, that they sometimes respond to personal repeated requests relating to minor issues. This potentially distracts them from their current task which might have a higher priority. On the other hand, other workers who observe this and are not treated the same way, feel neglected.

‘Yes, so if an employee comes to you, he may have already said something [about a specific error] twice or so. And you just didn’t react accordingly or told them to please follow this procedure. Sure, at some point there is a certain frustration. Of course, if we’re in a phase where we have time and can do something. Then you say okay, you know what, I’m just coming along with you, to take a look. And it can happen that it doesn’t work out the same for everyone and, well, then everyone interprets it a bit differently. It is quite normal. But our wish is that the process would go via a blackboard maybe, depending on what kind of project it is, rather than always going through backchannels.’

P06 and P07 – Maintenance

During the interviews, the installation of a large monitor on the shopfloor near the blackboard was discussed. Maintainers, however, fear that some long-term issues might cause frustration amongst the workers.

Another problem in this regard is forgetting of maintainers’ equipment. In some urgent cases, colleagues call them off their current task. In their hurry, maintainers tend to forget tools and to take their phone with them, which makes them almost unreachable in the meantime.

All this adds to the general frustration with respect to the lack of a consistent list of issues and the transparency about its status.

### Difficult prioritization of maintenance issues

Once the issues were reported, prioritization is the next issue that maintenance has to deal with. Most importantly, the lack of transparency of all current issues is one root problem, as there is no one source where all issues are being collected, followed by no consistency as to how and by whom priorities are set. As stated before, only important issues make it to the whiteboard in the remote shop-floor meeting area. During these meetings, even these issues are seldom discussed completely, if at all. Thus, only the most important issues are discussed and prioritized by all attendees.

During the rest of the shift, usually, maintainers are effectively accountable for prioritization and make their assessments based on the current situation individually or within the maintenance team. In-person requests are particularly powerful when providing assistance although the issue at hand might not be very important compared to the overall situation. Maintainers do not want to let their colleagues down or cause trouble on a personal level. Also, it is sometimes not clear how severe an issue might be, and they assess the situation in person. In these situations, maintainers also try to fix minor issues directly. Although maintainers are directly assigned to the head of production, they feel on a par with workers, foremen and shift-leaders, some of them being friends or neighbors. Consequently, they tend to avoid any unnecessary personal conflict and assess the priority of certain issues as higher than they otherwise might be. According to the maintainers, this gives them a permanent problem when weighing up their overall estimation of priorities.

The location of maintainers’ tasks is also an important factor for situated prioritization. Due to the size of the production site, maintainers tend to work on even unimportant issues close by, if they can be fixed relatively quickly, to avoid unnecessary unproductive transit times.

According to all participants, the lack of transparency of the entire maintainers’ workload (reactive and preventive maintenance, prototyping, etc.) is one of the most severe problems. Everybody desires a comprehensive and easily accessible list including the status of work, in contrast to the rather inflexible and remote whiteboard in the shop-floor meeting area.

There are different opinions about **who should decide about priorities in the future**. Maintainers do not feel comfortable with the current situation described above, but also foremen know that they cannot determine priorities due to the lack of access to the overall picture. This leads these roles to expect superintendents or even the head of production to make these decisions. However, superintendents and the head of production both only want to be informed about very important issues that severely affect the flow of production. Here, bottleneck resources are an important factor for them. These bottlenecks, however, depending on the current overall production situation which can vary from shift to shift, making even this distinction between important enough and unimportant difficult. Maintainers whish that all issues are brought to the shop-floor meetings by the foremen or others and then be prioritized collaboratively or by superintendents or the head of production.

‘This is a topic, it just has to be defined. And I think that this is a topic that has to be defined from above, from the production management down. To know that we have a priority here. That it’s okay for us to cancel any other project and get on with this new one, if it has priority.’

P06 and P07 – Maintenance

This procedure, however, is too inflexible for foremen, especially during the shift. Nevertheless, everybody agreed on the need for a specified means of communication that should be adhered to by everybody. However, there was no agreement to be found in the interviews as to what that should be. According to the head of production, the initial priority assessment of the worker is an important factor and should be considered in a future process. However, he suggested, it should be considered that this assessment should not become a very high priority, as it would dilute more fine-grained assessments during e.g., the shop-floor meeting.

### Brief summary

In this chapter, we have described in detail the various challenges that beset maintainers and the practice of maintenance and where they are encountered in the day-to-day work of the company in focus here. These challenges can briefly be summarized as 1) hurdles to the clear and timely communication of errors to maintainers as well as others such as foremen, which hinders the emergence of clear responsibilities (who is to address these errors first) which results in further difficulties such as unclear root causes or escalated errors which cause more severe production delays and are more difficult to repair. 2) There are currently insufficient procedures and tools employed to facilitate proper documentation of all tasks that maintainers need to be aware of. This results in maintainers forgetting some tasks but also prevents them from being able properly prioritize as neither them nor others, including workers and foremen, have full transparency over all tasks.

To begin to address these difficulties, as a next step we organized workshops with key stakeholders in the company and developed first sketches for possible interventions, which we will describe below in the next chapter.

## First iteration – workshop and sketches

Following the qualitative study, we organized four workshops, to work towards a final design. The first workshop served to jointly analyze and consolidate our findings and the following three workshops to iteratively evaluate early prototypes together. In this section we will briefly summarize this process and the outcomes.

### Analysis and consolidation—the first workshop

In order to jointly analyze and consolidate our findings with the staff of the company and to make design decisions that would enable the development of a prototype we organized a workshop. To the workshop we invited all the participants from the interview study. Most participated and discussed the findings, with only a few exceptions due to their heavy workload. To be precise, both maintainers, head of production, two masters, digitalization manager and some workers were present—all in all, 10 employees of the company. We firstly presented a summary of our findings from the interview study. After a short discussion two previously prepared design ideas were presented as sketches to inspire further discussion about how to proceed with the project organizationally and technologically. Based on the insights from the study so far, we had developed two different ways to support the intended (future) process to be implemented in the organization’s structure. In this first step we did not want to work on specific interface designs but provide a vision of a new process and an idea on how to support it. Our intent for these was not to prescribe design interventions but to inspire their development in these workshops, to provide graspable and concrete examples. Both technical solutions were independent of the organization’s infrastructure. We were explicitly asked by the head of production and the digitalization manager to implement a standalone solution (if a technical solution had to be implemented at all) and not connect it to any systems already in use.

**Figure 7.**
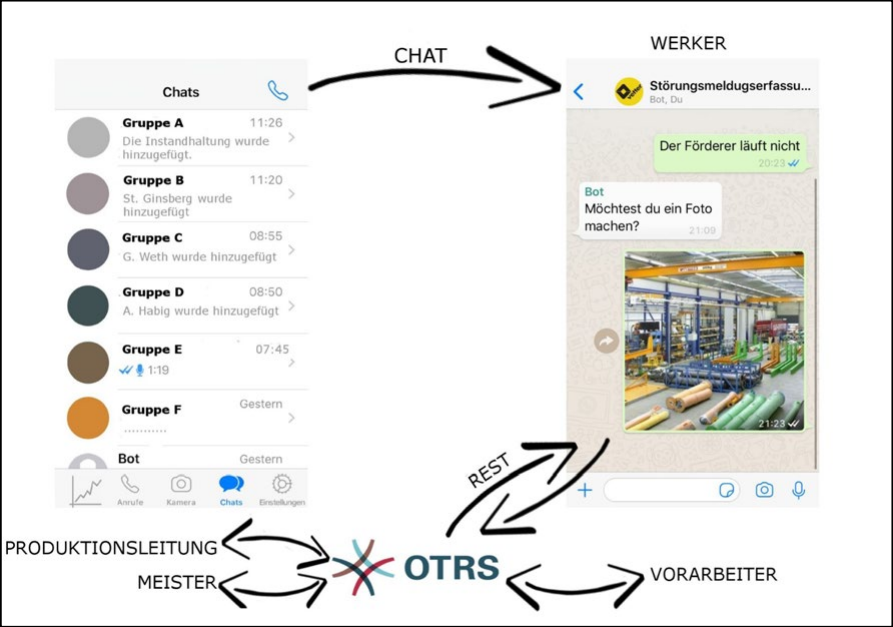
A smartphone- or tablet-based app for entering and managing issues. An optional chat feature should guide users through the ticket creation process

**Figure 8.**
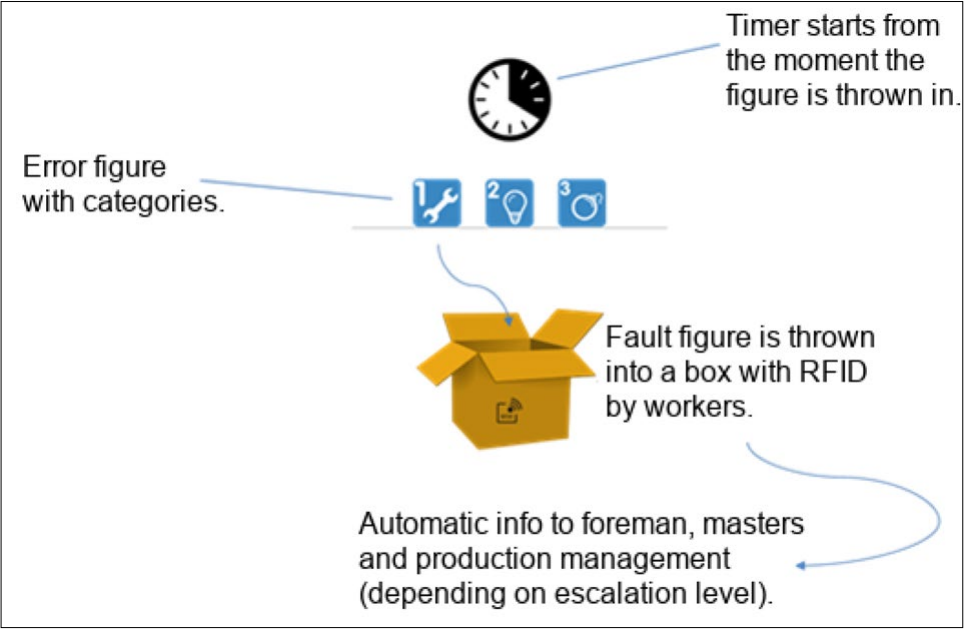
Technical solution where a token has to be thrown into a box to automatically create a ticket (or information in general) for maintenance and worker does not has to enter further information

The ideas we presented were 1) a ticket system with a chat function, which would be based on an existing open-source ticket system (OTRS; Figures 2 and 7) a system with physical RFID-based tokens to be thrown into a box to report an error, to reduce interaction time with any digital tool (Figure 8).

The discussion during the workshop unveiled four major aspects that any design would need to address: transparency about issues, prioritization of issues, the flow of information, and an IT-Helpdesk system.

Especially for foremen and higher management, transparency of all issues throughout the production – also outside the foremens’ field of responsibility – was one of the most important aspects. On the one hand, everyone should be able to see their specific error and that reporting has taken place. On the other hand, everyone should be able to estimate the importance of an issue in relation to all the other ones reported from other parts of production. Indications about the current status of issues were also desired, so foremen, for instance, might not need to contact maintenance for this info.

Directly related to the need for transparency was the issue of prioritization. Workers and foremen, but also maintainers desired that prioritization would be formalized in some way. As the process of reporting issues is a long-lived practice and much more intertwined into other processes as it seems from a first look, we never intended to fully capture it so that 100% of issue-reporting works with a design solution. Sometimes an unformalized way of dealing with issues like writing it on a piece of paper was within the realm of possibility and as we later saw, a practice from time to time. Also, prioritization should be made transparent, so everyone could evaluate the current ranking of a task against all other issues. During the workshop, different procedures to build priorities were discussed. Finally, participants agreed that each role from workers over shift-leaders and foremen, maintenance, superintendents and finally the head of production should be able to add or change the priority of an issue. The person entering an issue will do the first estimate based on three non-numerical categories of severity, time to repair and frequency. Based on the respective level a cumulative priority should be calculated.

Many participants preferred a formal procedure of reporting errors to the current informal one. Workers or shift leaders should inform their foremen (if available), who then notify maintenance directly. In urgent or severe cases, also superintendents should be informed, who would then inform the head of production in case of severe impacts on production planning. Despite this rather complicated procedure, participants also voiced the importance of receiving feedback quickly, that their issue has been noted by maintenance. Furthermore, they also wished to see the progress of any specific issue to estimate its completion.

Finally, and after a member of the IT department demonstrated an existing ticketing system in use at another department, participants decided on a ticketing system (Figure 7) as the most feasible option to achieve the requirements above, for which existing tablets could be utilized. In the past, a ticket system was not in use for the participants of our study, so it is quite unlikely that they had extensively worked with such a system.

### Concept and implementation

Building on the insights from the study and the workshop, a low-fidelity wireframe prototype based on Microsoft PowerPoint was created, that demonstrated basic the user interaction of a ticketing-system. This prototype was presented to the head of production, both maintainers and one superintendent. Their feedback was then used to create further versions.

For organizing and prioritizing tickets, the KANBAN concept from software engineering was adapted. The central visual element, the KANBAN board, became the main tool for maintainers and higher management to prioritize tickets and change status. In KANBAN, there is a column for each status, containing all tickets accordingly. Tickets are visualized as cards that can easily be moved up and down, thus changing their priority, and across columns to change status. KANBAN follows a ‘pull principle’ so that maintainers in this case choose which tickets they take on next, depending on their situated assessment.

Four statuses of tasks were chosen: worklist/backlog, in progress, paused and done. All new tickets are automatically assigned the status ‘worklist’.

**Figure 9.**
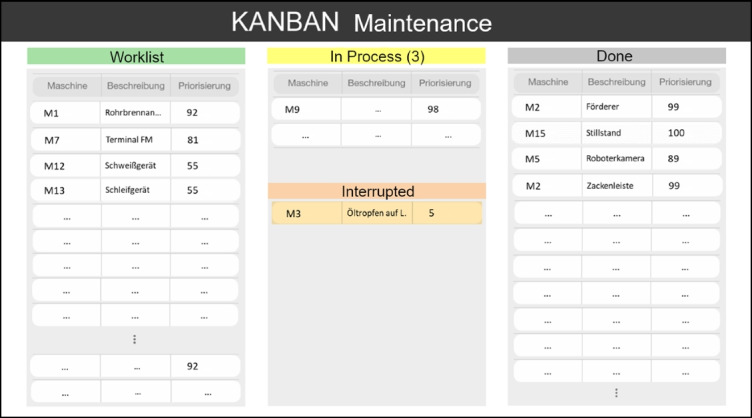
KANBAN board, expected to be the main view for maintenance

The visualization of tickets on the board should assist maintenance as well as higher production management to organize the tickets (see Figure 9). The view is separated into three columns where the middle column is separated in two on top of each other, representing the four statuses. On the left side is the worklist, in the middle ‘in progress’ on top and ‘paused’ below and finally finished tickets on the right side. Each ticket is represented as a small card, only showing the affected machine, a short description and the priority as a number. Cards should be moved to change its priority or to change its status, so other colleagues such as foremen can see that changes were made.

**Figure 10.**
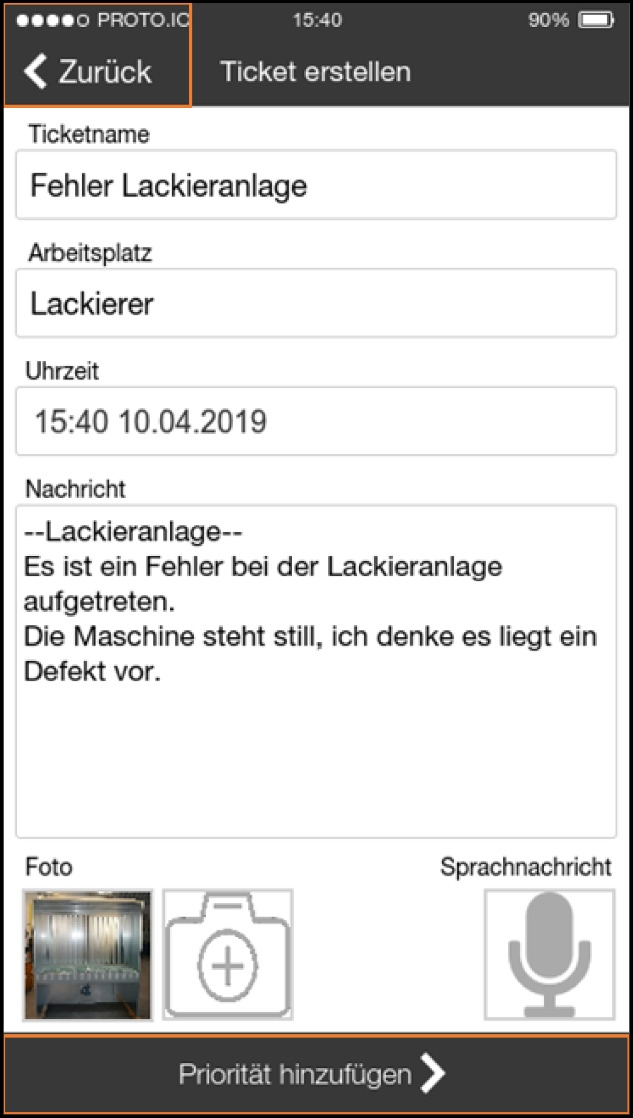
View where meta-information can be entered for the specific issue

**Figure 11.**
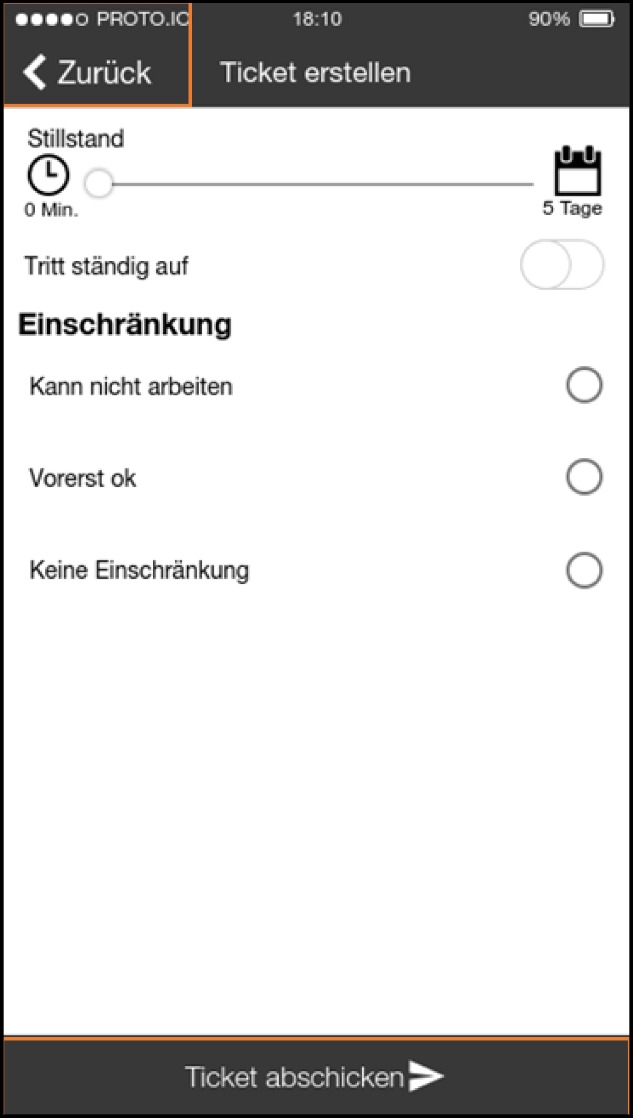
View where workers can prioritize through explaining how critical the issue is

**Figure 12.**
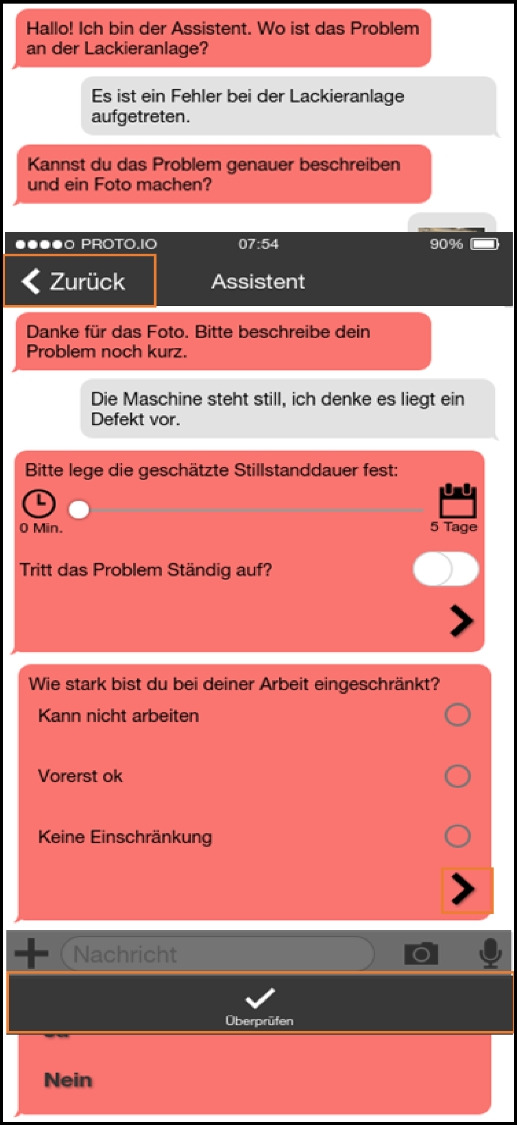
A chatbot should provide a guided instruction for creating a ticket

**Figure 13.**
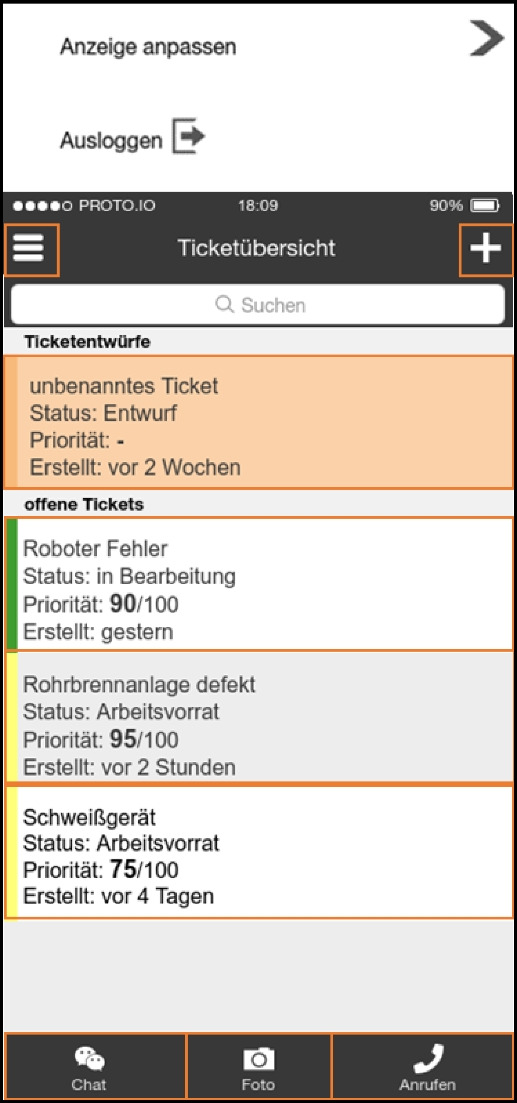
View for foremen where all tickets from their production line should be listed

Creating tickets should be made as effortless as possible but gathering all necessary information for maintenance. Thus, a two-step process was chosen. First, general information about the issue should be entered, with title and workplace as mandatory fields and an optional field for further description. Also, pictures or audio records could be added optionally (see Figure 10). In a second step, an estimation regarding prioritization should be made, also optional (see Figure 11). The person entering the ticket should estimate the severity of the issue (using the categories of ‘can still work’, ‘can work with a workaround’ and ‘cannot work anymore’), time to repair and frequency of the issue. These non-numerical values are then calculated into a priority that can reach 70 of 99 points maximum. This threshold was set, to leave space for further prioritization by maintainers, supervisors, or the head of production. As an alternative way to enter tickets, a chat-based interaction should be implemented, guiding users through the process by explaining in a little more detail the need for the very same information (see Figure 12). For an overview, tickets should be displayed according to priority, with most urgent tickets on top (see Figure 13).

Throughout this part of the process, three workshops were organized to gather intermediary feedback on the design from the practitioners. In these workshops, participants made several requests for improvements. For example, it was deemed desirable to have several views of the board, with visibility and transparency increasing with hierarchy, so that workers see only tickets regarding their workplace, but foremen, superintendents etc. have a gradually greater overview over current maintenance tasks. Participants also requested specific filtering mechanisms, such as filter for workplaces. Maintainers requested the ability to enter their estimated time to completion separately. It was also decided that users should not have individual accounts, but that shared accounts according to role and workplace would be sufficient. Lastly, it was also decided to remove the whiteboard that served to document the morning meetings with a screen to display the KANBAN board.

## Final concept and design

During this process we gradually worked from a MS PowerPoint-based wireframe towards a fully usable implementation, based on the OTRS Open-Source tool mentioned above. The final concept x and the implementation that was developed are described here, using screenshots of the final prototype.

### List and detail view

**Figure 14.**
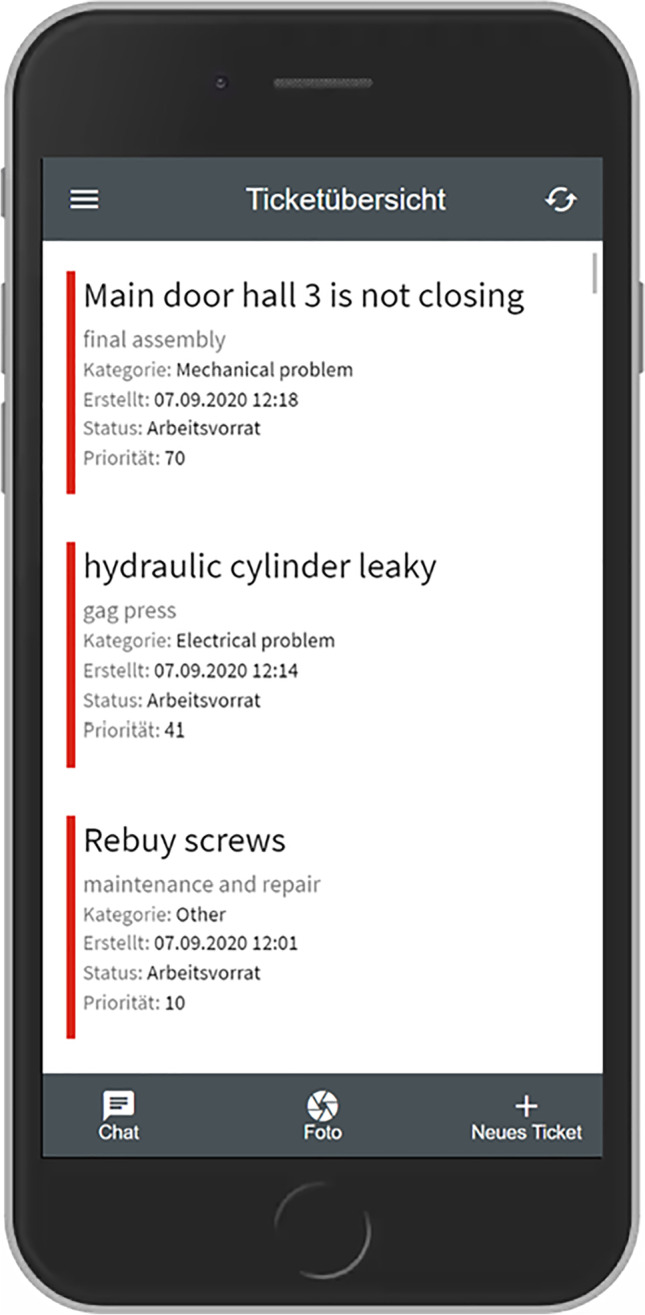
List view

**Figure 15.**
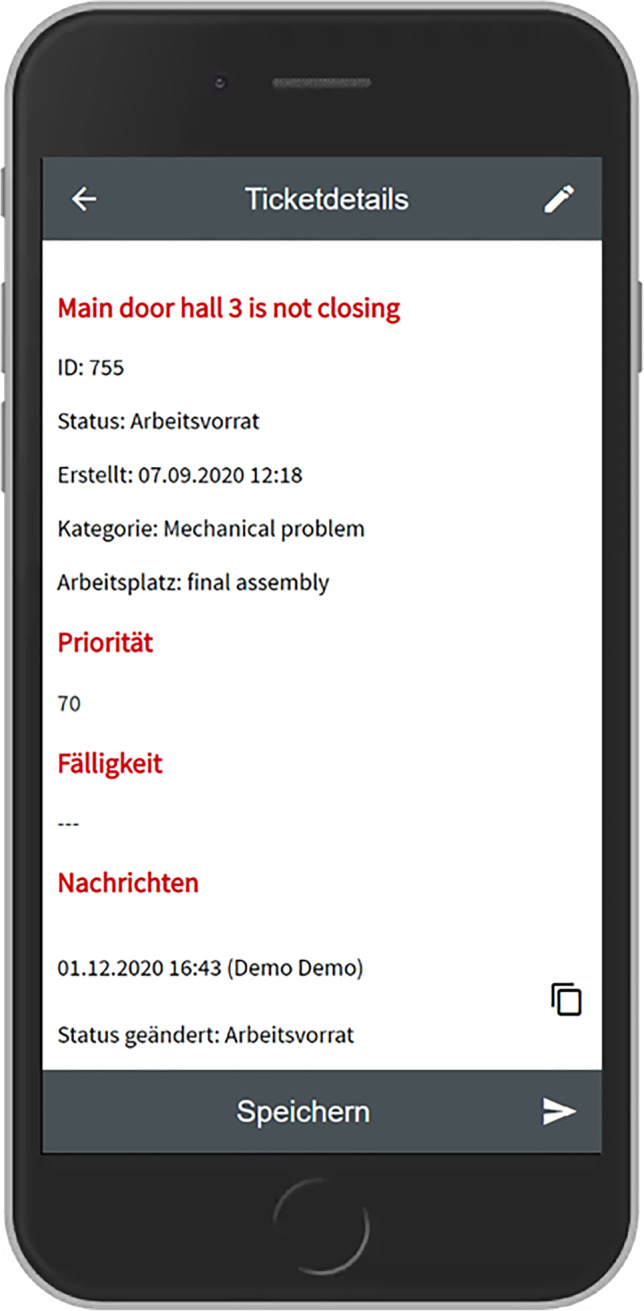
Detail view I

**Figure 16.**
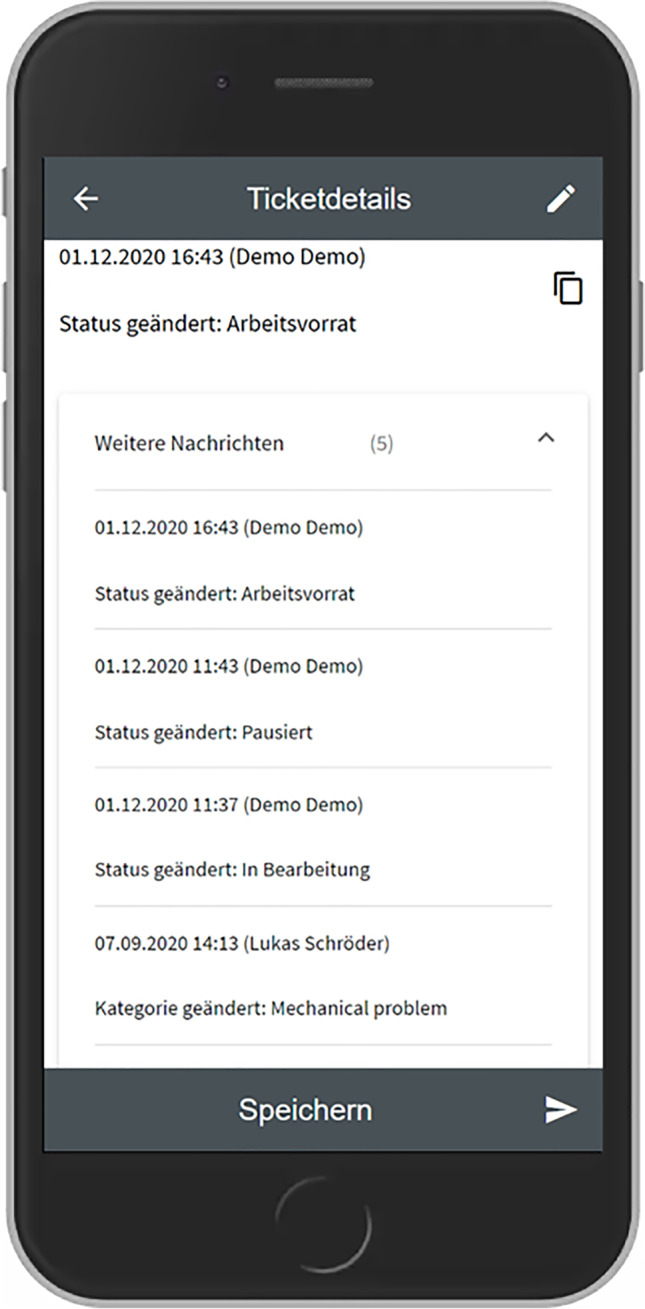
Detail view II. Descending message log

The list view (Figure 14) was intended to be used mainly by foremen and shift leaders and later also by workers, to show only tickets from their field of responsibility. Although participants initially wanted to see their tickets matched against to all others, this way of representation was selected to keep a better overview of daily business. The list is sorted by date descending, showing the most recent tickets at the top. The colored bar at the left side indicates the status of the ticket (worklist, in progress, paused, done). The title of a ticket is intended to contain the most important information about the ticket. Additionally, the workplace, category resp. reason for failure, creation date, status and priority are outlined. Further information can be accessed by tapping on a ticket, opening the detail view (see Figure 15). In this view, workers and foremen can only view the ticket information, whereas all other roles can make changes to all fields, add more pictures as well as write messages to the ticket that are presented in a descending list (see Figure 16). For every change of status or priority, a message is created automatically, noting the user, time, and kind of change. Thus, transparency about such changes should be provided to everyone interested, without any further effort by those making the change.

### Creating tickets

**Figure 17.**
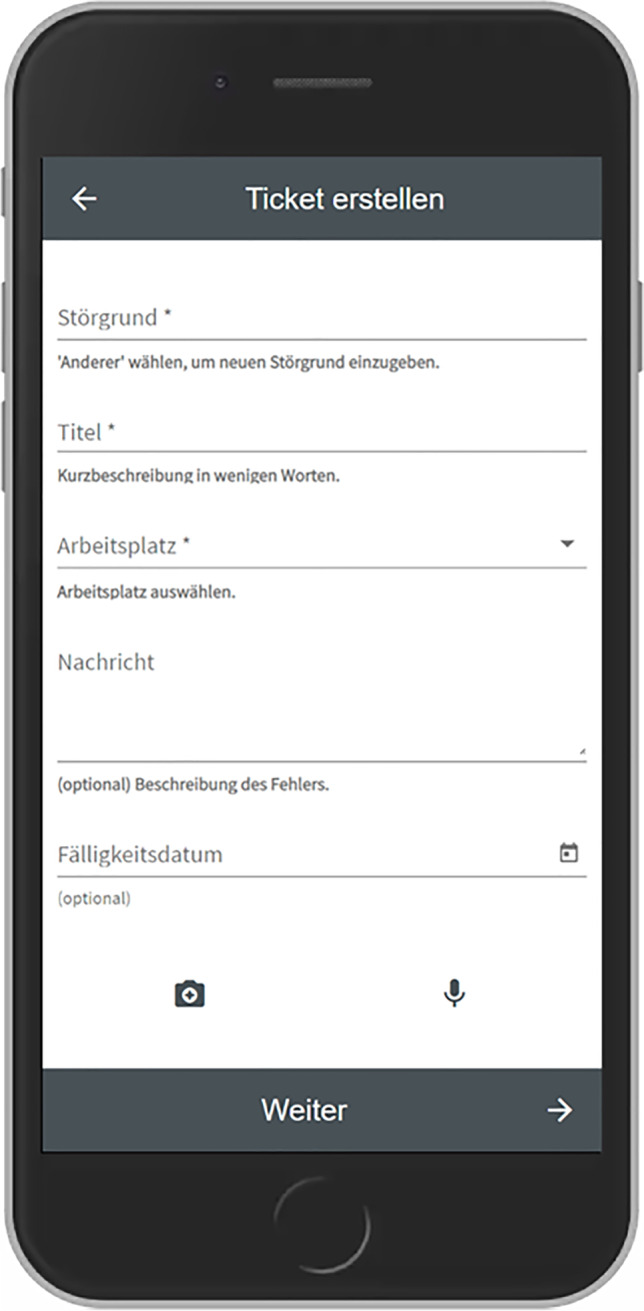
Create ticket – general information

Tickets can be created in three different ways, by using a regular form; by taking a picture or assisted by a chat (see Figure 14 bottom). The regular form was the preferred way of entering tickets by the participants (see Figure 17). However, there were concerns those users who were not involved in the design process may have difficulties understanding the purposes of each field e.g., how priority should be set. The idea of a chat-based assistant to guide users through the process by asking questions that explain the need for information and add more detail to its purpose, was provided as an alternative. For example, the different sliders on the prioritization view are decoupled into single ‘messages’ and are introduced with a sentence which provides some information to the user on what this specific component does. The picture mode simply starts the process by first taking a picture and then forwarding the user to the regular form, asking for completion. The idea is that users first take a quick picture of an issue with the camera on the mobile device and complete the ticket elsewhere.

**Figure 18.**
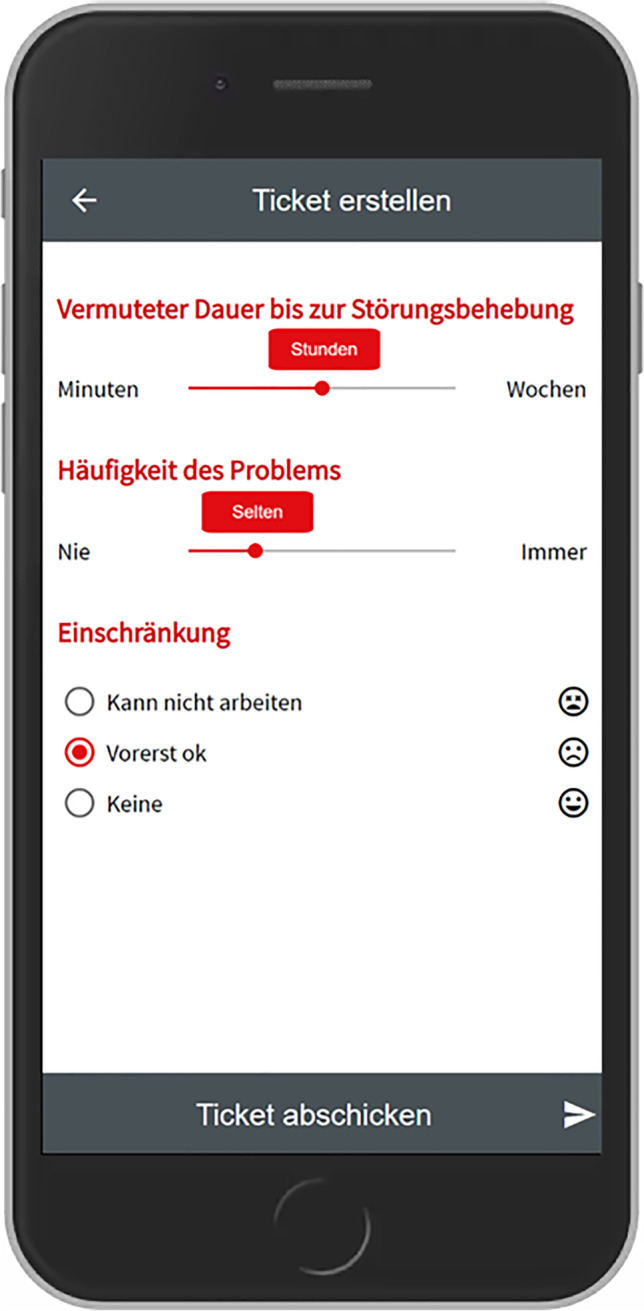
Create ticket – time to repair, frequency and severity

**Figure 19.**
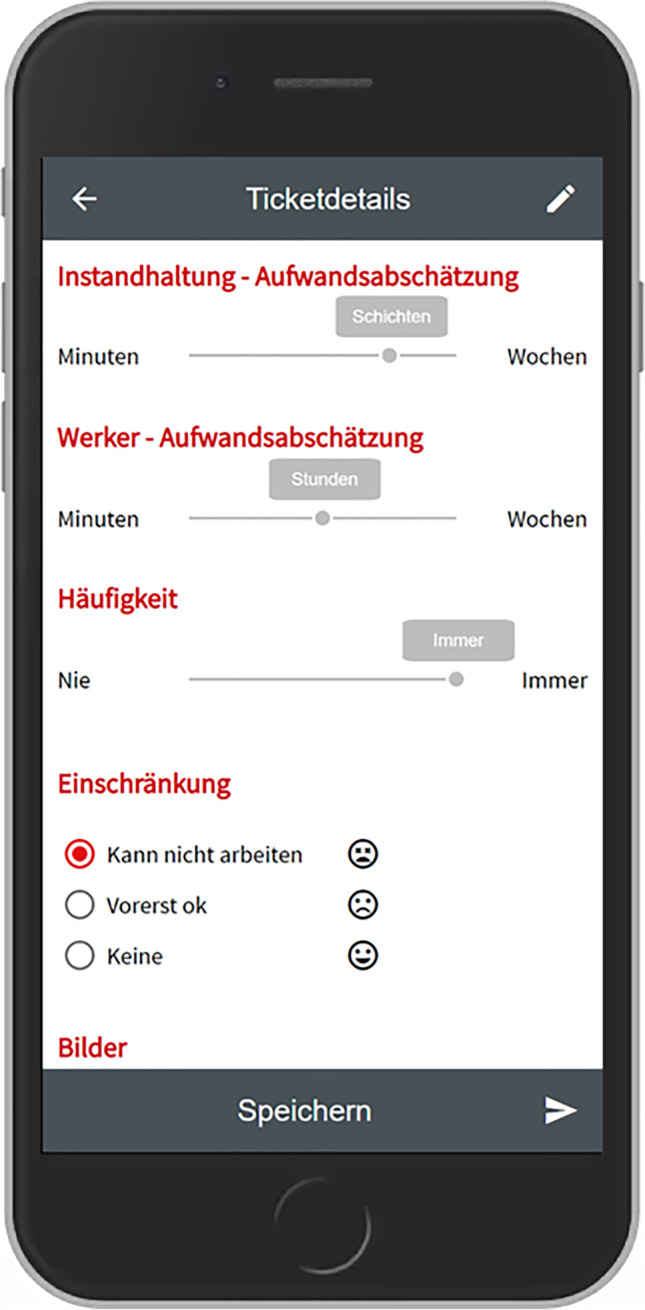
Ticket details also showing maintainers’ estimation

Entering a regular ticket is done in two steps. First, general information such as the mandatory fields ‘reason’, ‘title’ and ‘workplace’ must be entered (see Figure 17). Additionally, a description can be entered as well as a due date. The latter is intended to be used for projects that maintainers also have to work on or other tickets that need to be finished at a certain time. Also, pictures or voice messages can be uploaded, using the camera of the smart device or file dialogue on a PC. Pictures thus can act as a supplement to the text fields. Voice messages also support users who do not want to write or when writing much text is too much effort or not possible in a certain situation. On the next screen, the user is asked to estimate the time to repair, using a slider showing attributes ranging from undefined, minutes, hours, shifts or weeks (see Figure 18 and Figure 19). These attributes are thought to be answered more easily and accurately than estimating a number from a given range. Similarly, users can estimate the frequency of an issue, ranging from never, rarely, frequently, regularly, and always. Finally, an estimation of the severity of an issue should be provided. The users can select from the options ‘no influence on operations’, ‘ok for now’, indicating that maybe a workaround is in place and a final solution should be found soon, and finally that a ‘user cannot work anymore’.

The discussion during the workshop had a big impact on this design decision as the participants agreed that it would not be suitable to estimate a clear number to determine the exact priority (like is it 56% problematic or 57%?). A deep discussion on prioritization led to these three categories (duration, frequency, severity) which were then implemented as shown.

All these estimations add up to a priority of maximum 70 out of 99, leaving the remaining range for maintenance and higher production management for their own reassessment. It was intended, that in the morning meetings or during the shift, superintendents, the head of production or maintainers could change priorities manually, bringing all tickets in an order with regard to the overall situation on the shopfloor (Figure 19).

### Organizing tickets – the KANBAN view

**Figure 20.**
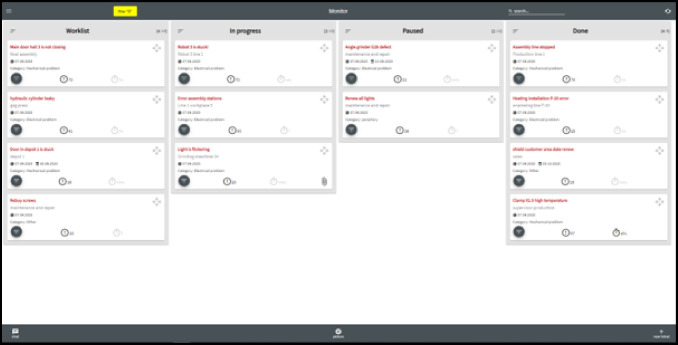
KANBAN view as the main view for maintenance

**Figure 21.**
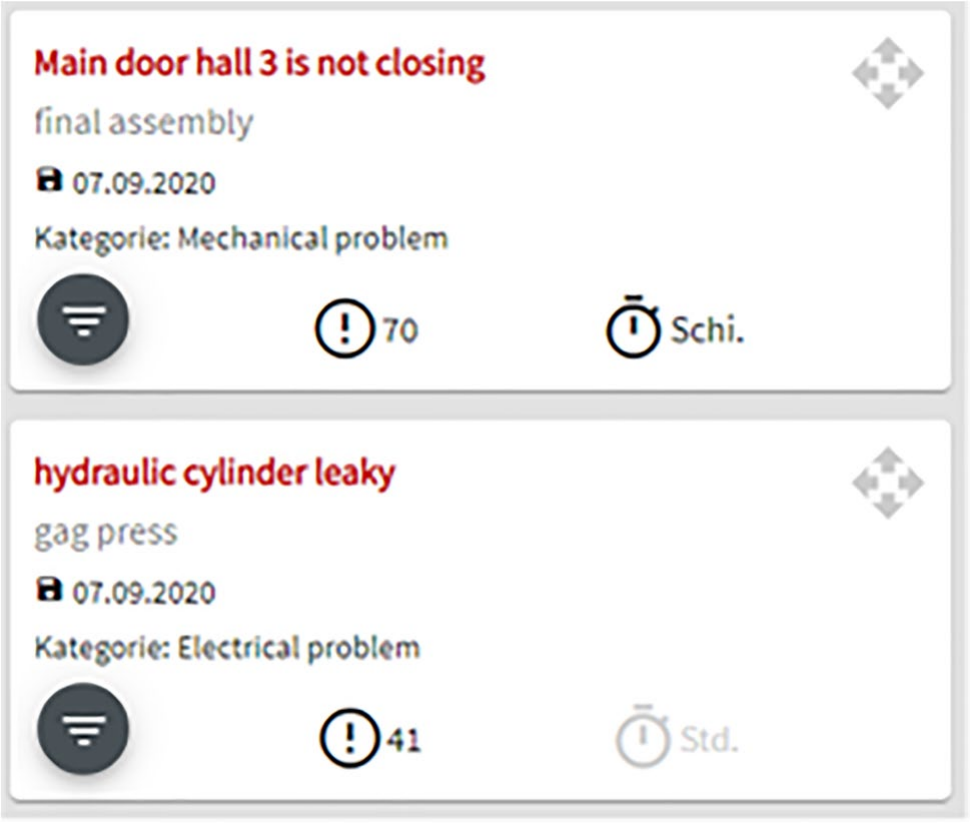
Tickets in the KANBAN view contains suitable information for maintainers

For supporting maintenance in organizing their tasks and managing priorities by all other managing staff, a KANBAN board view was created (see Figure 20). This view and interactive interface follow the KANBAN pull principle. The main elements of the board are the columns representing the state of tasks. During the design process, four states were selected: ‘worklist’, ‘in progress’, ‘paused’ and ‘done’. As mentioned before, all new tickets get the status ‘worklist’, which is like backlogs in software development. The column is sorted descending by priority, but can be changed the attributes workplace, category, or due date. This way, the most important tickets are shown on top, to be recognized more quickly and conveniently. Tickets are then pulled to the next columns, typically ‘in progress’, to indicate that these tickets will be handled at that moment or any time soon. Dragging and dropping is done by tapping or clicking and holding the cross at the top right of a ticket card (see Figure 21). The design of the ticket cards was created based on the first evaluation outcomes and contain the most important information for getting an overview of the topic, location, creation date and priority of a ticket. Also, the estimated time for repair by foremen or workers is indicated (Figure 21; light grey stopwatch). Maintainers can also set their own estimation using a separate slider (see Figure 19; top slider). The color of the stopwatch is then changed to black and showing the new estimation. The previous estimation remains and can still be accessed through the detail view (see Figure 15).

This view was mainly inspired by two indicators based on previous knowledge and our findings during the interviews: As the maintainers, head of production and other roles use a whiteboard in their daily updates every morning they were accustomed to a tabular view where every incident is on one row or, in our case, a card. Also, the connection to the work of IT-departments was quite close and we ourselves had used a ticket system a lot in the past. As maintainers’ work was somewhat ‘not plannable’, a pull-principle seemed like an adequate solution.

During the workshops, the KANBAN process was described, and the participants consistently argued that maintainers should decide which specific ticket they should work on next. The order by priority of the first column should only be an orientation for maintainers, leaving it up to them which tasks they will take on in any given situation, based on their overall assessment. The column labelled ‘paused’ was created to indicate that an issue cannot be worked on at the moment, e.g., because of missing spare parts. Once a ticket is in place, it can be dragged to the respective column or by changing the status in the detail view. It was decided that closed tickets remain visible on the board for two weeks. After each interaction, whether a ticket was moved or changed in detail view, a respective entry is made as a comment to the ticket, so other users can follow its progress. Each of these messages contains the message text, creation time and username (see Figure 16). This, it was felt, should provide enough information without any additional effort for e.g., maintainers to satisfy information needs by workers or foremen.

### Filter

**Figure 22.**
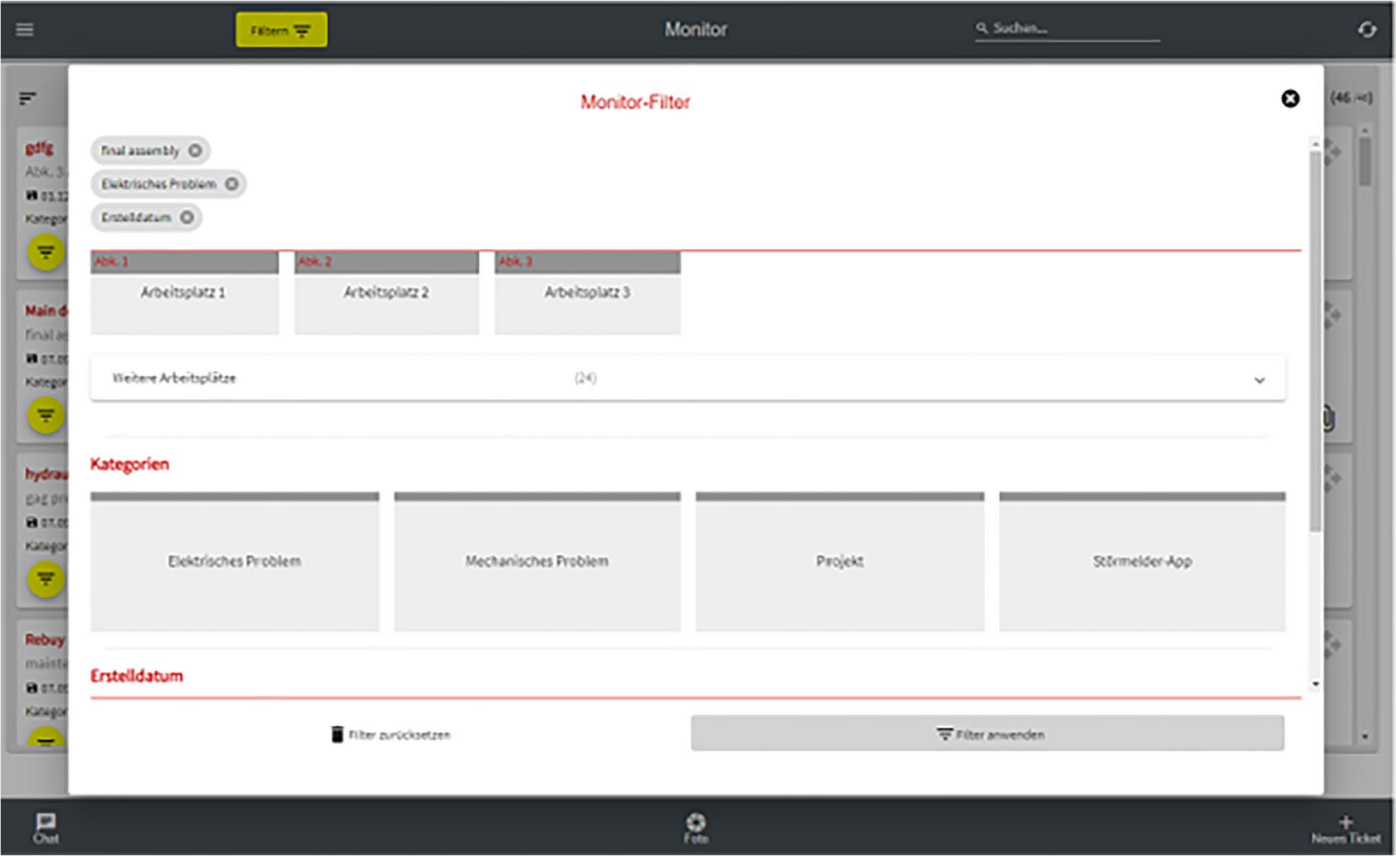
View with filters to quickly find tickets of specific type, with specific name or workplace

For supporting fast information retrieval, especially during the shopfloor meetings with its very limited time per participant, a filter-view was created. Users can filter by workplace, category, creation date and due date. Standard workplaces are presented as visible and selectable cards on the view, whereas custom workplaces are summarized in an expansion panel that can be unfolded to list all other workplaces in the same way (see Figure 22). Additionally, via the search bar at the top of the KANBAN view all tickets are being searched in full text. This can either be used to search for a specific ticket or to filter tickets when e.g., workplaces are known. For a very quick way of filtering tickets by workplace, a filter button is placed at the bottom left of each ticket card. By toggling the button, the lists are filtered for this exact workplace. This feature is meant for foremen or maintenance to be able to report quickly about further issues regarding a single resource.

### Socio-technical implications

Apart from designing the software for supporting maintenance coordination, complementary organizational measures were implemented. Input from IT support, from the project leader of the company as well as from the researchers, led to an agreement on a new practice which meant that oral ticket reports could still be stated, but co-workers then have to create a ticket using the tool. This, it was thought, should gently push those who prefer to report directly to the maintainers towards making further efforts to organize the workload.

Also, the head of production decided that the tool should first be tested by maintainers and foremen to rule out bugs and to ensure they were familiar with the tool. Another reason for this partial rollout was that there were not enough old iOS devices to provide for every member of the production team. Foremen, however, as mentioned before, already had been using tablets for their daily work, so it was easier to start this way. Maintainers were equipped with spare devices first, as they had not used such devices before.

### System rollout

After the tool reached a relatively mature state, it was first tested by the maintainers in order to identify usability issues and other concerns that might emerge during its use. The researchers and maintainers agreed that a meaningful test could only work if at least all aspects of the whiteboards were entered into the system. It seemed that maintainers would not do this any time soon due to their daily obligations, so the researchers took on this assignment.

After this test phase, the tool was updated several times based on users’ feedback. The head of production decided that the rollout of the new tool should happen during the shopfloor meetings. After consultation with the researchers, it was deemed by the head of production that their presence was not necessary. The first users of the tool were the foremen of one of the two production lines, maintainers, superintendents, and the head of production. Workers, shift leaders and foremen of the second production line should be included later after the tool was further evaluated by the staff and improved accordingly by the researchers.

## Situational evaluation

To understand how the tool was used in the company and how it affected maintenance practices, we opted for a situational evaluation approach. A situated evaluation (Twidale et al., 1994) took place. This is intended to deal with the inherent difficulties of evaluating CSCW systems outlined by Grudin (1988). These include the effects of the introduced system on the behavior of other group members, the influence of social, motivational, economic, or political dynamics on the appropriation of the system as well as the importance of time, as use of a system might change over days, weeks, or months. In our case this meant for example that the tool did not only affect the work of the maintainers, but also other members such as operators, foremen, superintendents and so forth, and that the introduction into the organization resulted in changed work practices which in turn affected the use of the system. In accordance with CSCWs commitment to studying practices in situ*,* which our own work shares, situated evaluation rejects the view that evaluation necessarily takes place at a specific moment of the design process and is not to be seen as summative. Rather than validating the usefulness of our tool, we sought to identify common experiences by users.

The evaluation of the tool took place on two occasions after the official rollout in October 2019. The first evaluation took place immediately two weeks after roll-out, the second evaluation one year later in October 2020. Each evaluation included a visit to the production site by the researchers, observation of use practices of the tool and interviews with the head of production, two maintainers, one worker, one foreman and the project coordinator (see Table 1). Unfortunately, in the second evaluation, we could not talk to all the employees we had previously interviewed due to the COVID 19 pandemic and increased workload at the production facility.

Although the researchers offered their assistance for the first rollout, the company representatives decided to do this themselves during one of the shopfloor meetings. They had become so familiar with the tool that they felt comfortable introducing the tool to the production line without any assistance by the researchers.

The following evaluation is divided into two sections. First, a quantitative analysis of tool usage is presented, to provide an idea about the intensity and manner of usage. Second, the results of the two evaluation workshops are presented.

### Data evaluation

**Figure 23.**
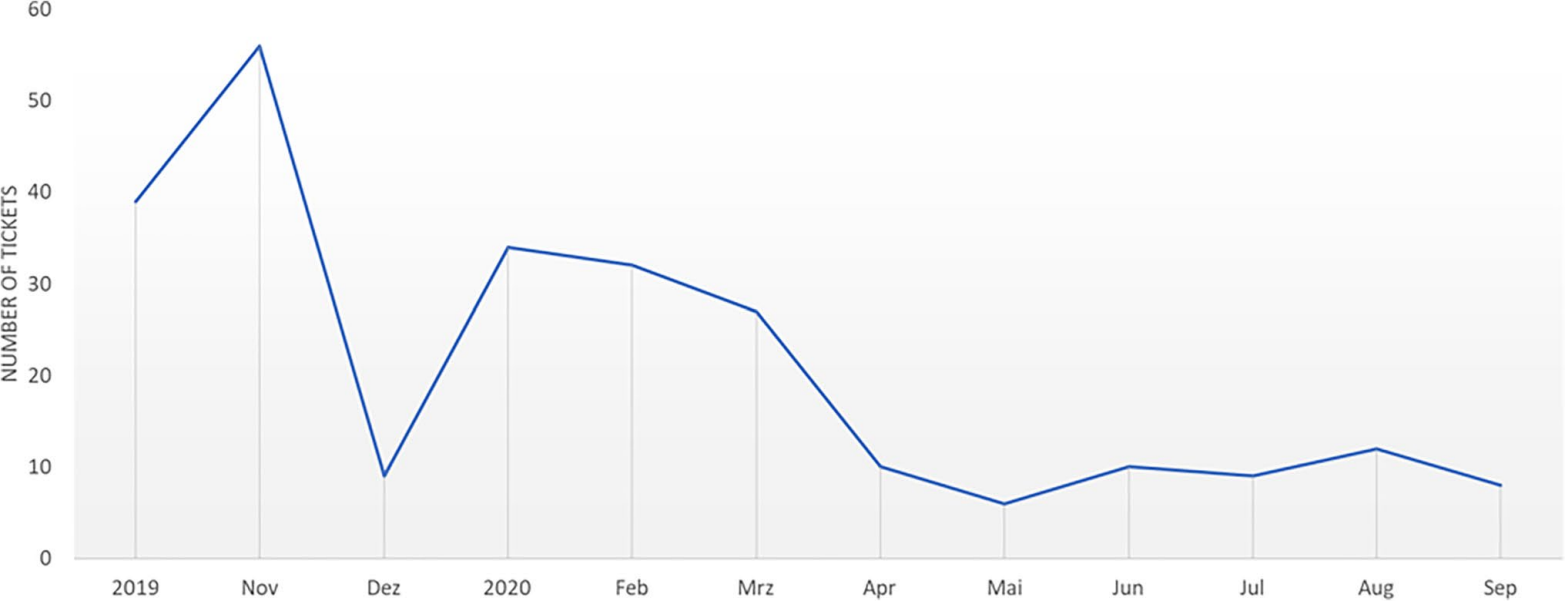
Dissemination of the number of new tickets per month during the evaluation period

During the evaluation phase of one year, 252 tickets were created in total by user accounts in the company. As shown in Figure 23, most tickets were entered at the beginning phase, between October 2019 and March 2020. From April until the end of September 2020, the number of new tickets reached a rather constant level of approx. 10 new tickets per month. At the beginning of the evaluation phase, the researchers entered all issues previously on the whiteboard into the system, which explains the high numbers at the very beginning. However, during the first month, many unresolved minor issues were entered, before a relatively normal and constant level of new tickets was reached. The decrease from March to April 2020, however, coincides with the first lockdown due to the COVID19 pandemic in Germany.

**Figure 24.**
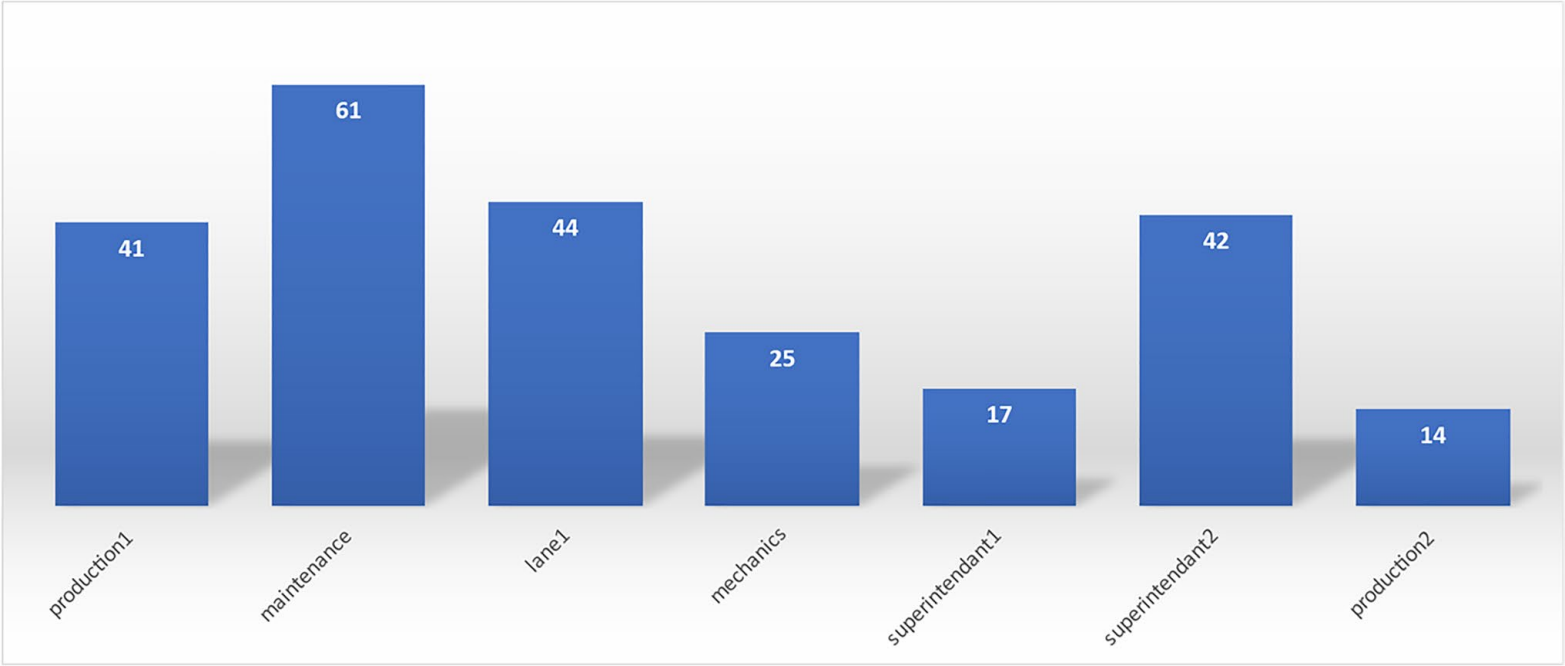
Total number of created tickets per user account

Most tickets were created through the account of the maintenance department (see Figure 24). This number, however, includes the initial 30 tickets entered by the researchers at the beginning of the evaluation phase. Surprisingly, superintendents entered 59 tickets, although they were not mentioned as a highly active part during the workshops (see 8.2). All other accounts are used by foremen and most tickets were entered with these accounts. The missing eight tickets were created by the researchers’ and project leader’s accounts and are not listed in Figure 24.

As mentioned earlier, maintenance work can be either planned or on-demand and although the number of tickets entered and later added to by different roles in the company, we cannot definitely say whether maintainers work is more demand-based or planned.

**Figure 25.**
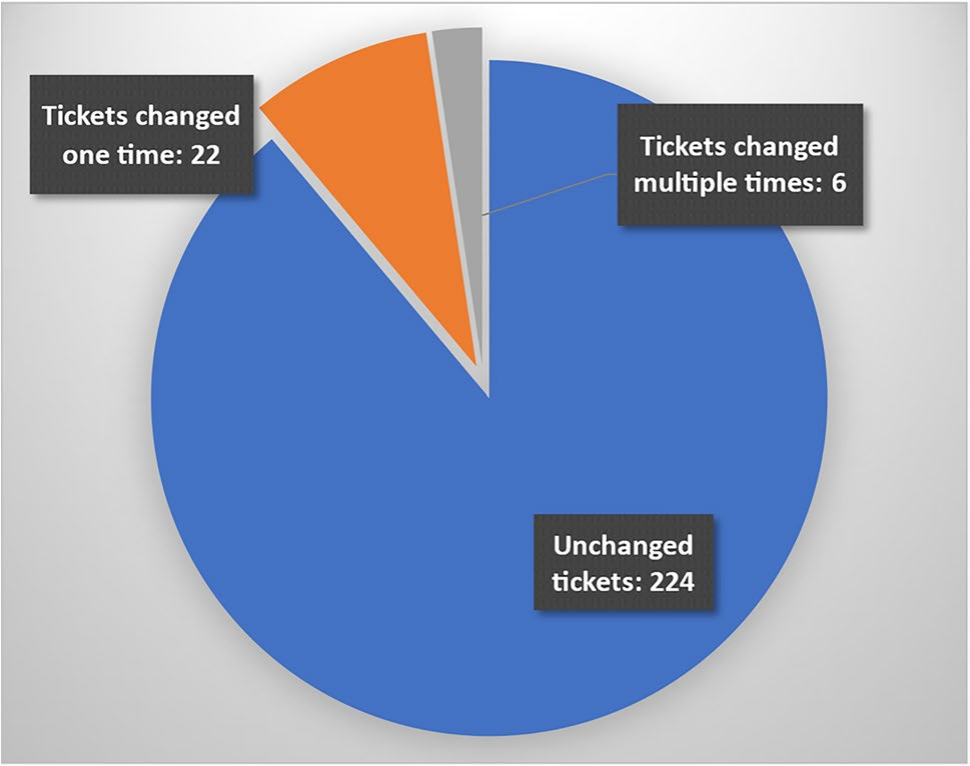
Number of changes in tickets after initial creation

Only eleven per cent of the tickets were re-prioritized after they were first issued (see Figure 25). This indicates that the formally intended way of negotiating priorities on a managerial level did not occur, but rather the first estimate by those users entering the tickets remained.

### Evaluation workshops

The design was carried out on the premise that workers should be the ones creating tickets, as was agreed upon in the earlier stages of the project. The intended effect was that foremen and superintendents could gain awareness about occurrences in their field of responsibility and maintainers could see and react to new tickets directly. The need for phone calls should be reduced and the status of tickets made transparent in the system. However, at the first evaluation two weeks after rollout, as well as during the second evaluation one year later, it became clear that the tool was not being used at the worker level but remained in the purview of the original user group of foremen, maintainers, superintendents, and the head of production.

#### New issue-reporting practice

Foremen stated that they are satisfied with the new tool and appreciated the new information flow and their enhanced awareness about tickets and their status. The information flow now is more in line with what they expected it to be even before this project.

Maintainers mostly adhered to the new procedure and asked everyone who contacted them in person to create a ticket, so it would not be lost, referring them to the new tool. Workers then mostly contacted their foremen to report an issue. Together, they entered a ticket, using the foremen’s tablet. Often, foremen added a photo to the tickets to give maintainers as comprehensive a picture of the situation as possible. Sometimes, however, maintainers also entered tickets when contacted in person, if the new formal procedure did not seem appropriate, in order to speed up the process or to avoid potential tension. According to all participants, there is now a general understanding that only tickets that are entered into the new system are handled promptly. Maintainers, as well as foremen, emphasized this during the evaluation, which indicates that accountability for handling tickets is now distributed, where one party is responsible for providing the initial information and the other one for processing and updating information. The maintainers stated that to convince their colleagues to follow the new practice, they try to react to new tickets quickly. The tool is always open on their tablet or PC, and they check several times for new tickets per day and try to fix minor issues as soon as possible as well, to demonstrate that they too are recognized. However, in very urgent cases, such as machine breakdowns, maintainers still react immediately, even if no ticket has been created and even if the request does not come from any of the foremen.

Creating tickets is mainly done by using the default form as described above. Other ways of creating tickets like the chat-based interaction are not used regularly. As the participants mentioned, the process is rather simple and so a guided workflow is not needed for now. They also mentioned that the chat might be good in sending users questions about the state of an issue like if it is solved or other circumstances appeared.

Maintainers do not take their new smart devices with them, because they do not have a pocket with which to carry the tablet and do not want to forget the device somewhere in the production area, lose it or even break it. Thus, they return to their workplace regularly to update tickets handled previously, check for new tickets, and then select and note down on paper those ones they could accomplish next, based on priority and location, so even unimportant issues along the way can be quickly dealt with.

Tickets, then, are not always created afterwards. During ticket processing, when changing status or priority, maintainers leave notes, e.g., if spare parts are missing, or add pictures. Foremen found this information or the automatic notes about status and priority changes sufficient in most cases. As a result of this new practice, maintainers felt that phone calls and discussions about tickets had decreased significantly.

#### Established practice and tool support

After the tool was rolled out within the intended line of production, other foremen, from another production line, quickly adopted the tool, too. This first became evident, as the number of pre-configured workplaces (12) was quickly extended by 34 other workplaces. Many of these workplaces could be identified as regular workplaces in other departments. The rest were other locations such as the main gate or periphery. Foremen stated that their colleagues asked them to present the new tool and how to apply it, after seeing it during the shopfloor meetings. This quick distribution throughout the whole production site was facilitated by the availability of the right equipment (tablets with an internet connection), access to the platform that is accessible through the internet and non-user specific as well as generic accounts that were set up before and known to the project leader at the company.

During the evaluation period, the tool was used on a daily basis, but mostly by foremen and maintainers, and became the preferred tool for handling issues. According to the participants, there were no workaround tools in place. For a few, mostly very important issues, the tool was bypassed completely, as mentioned before.

Although the KANBAN view was intended to be the view for managing tickets, the maintainer who participated in the workshop stated that he preferred the simple ticket list, mostly to check for new tickets. Changes to priority or status he enters using the edit mode in ticket details.

#### Prioritization

The data analysis showed that prioritization in the system was mostly done by the foremen entering the tickets (see 8.1). These priorities were only changed during the biweekly meetings with maintenance and the head of production. During the meeting, maintainers made changes to the tickets with their user account based on the discussions which took place. In these meetings, the general maintainers’ workload is discussed, and new prototypes and other projects are planned and prioritized based on the overview given through the new tool. Predominantly, however, priorities were not changed after ticket creation.

Maintainers do not work off the worklist exactly by priority as presented in the tool, but make their own estimation based on the current situation and the information that was provided in the shopfloor meetings. However, maintainers stated that maintenance issues were not discussed in any more detail than before and that the format of the shopfloor meetings stayed the same. The large screen display that was bought for the purpose of discussing and handling tickets in these meetings is hardly ever used. As mentioned before, priority changes are only rarely made, so the maintainers evaluate their next assignments based on the priority provided in the system, the location of the issue and the overall picture they obtained during the day. Foremen were mostly satisfied with the way issues were dealt with by maintainers and appreciated the updates manually or automatically in the tickets (see also the citation in 8.2.5).

#### Transparency

Foremen particularly appreciated the overview that they gained through the new practice supported by the tool. This involves changes in tickets directly related to their field of responsibility, as well as those outside this area. In the second workshop, a foreman stated that he reacts to tickets from upstream workplaces: Due to the transparency of issues on every workplace, production line, or department, foremen became much more informed on ‘what is going on’. A foreman is then able to distribute the workforce between different production lines, tasks, or orders which in turn supports the overall efficiency (e.g., reduce the lead time of orders). As an example, one foreman mentioned that he can assign welders to other workstations when he sees that a breakdown might affect a workplace. As the company handles heavy and big parts consuming time and space to handle between workstations, this development enables the foremen to be better organized in their shifts.

#### Remembering undone work resulting in a satisfied work environment

Both foremen and maintainers emphasized that they appreciate the tool, especially for minor issues. Such tickets were often forgotten in the past and are now dealt with more consistently and frequently. As stated before, maintainers try to solve minor issues quickly on their way elsewhere. From the maintainers’ perspective, the foreman also stated that due to the ticket description and particularly the pictures, maintainers can now bring the right tools directly and thus save unnecessary journeys through the production hall to get the right equipment.

‘I love the app, I honestly have to say. In terms of production, everything that’s a minor issue, everything that remained undone, whether it’s a broken plug, a leaking connector, that’s taken care of now on the fly. They [the maintainers] pass by on their way to the mechanical workshop and a plug is broken on the firing machine, then that’s taken care of right away. The small things that used to be postponed because they were unimportant and only were reported on call, used to be forgotten and are now taken care of promptly.’

P11 – Foreman

## Discussion

### Reflection of the evaluation

Several of the comments made to us in the period of our evaluation suggest the significant effect the intervention had on the maintenance practices of the company and that these changes were welcomed. In the following, we will reflect briefly on these desired effects and changes, also in comparison to the requirements identified in the joint workshops at the beginning of the project. Nevertheless, the intervention also had unexpected and even surprising consequences during its appropriation, which we also discuss.

The workshops and our early investigations had revealed several pain points for the maintainers and other members, and they expressed a number of needs to be met. The most pressing ones were an improved flow of information, that would for example limit interruption to maintainers’ work; reducing the risk of forgetting; increasing transparency over the issues maintainers worked on, and an improved negotiation of the prioritization of maintenance tasks.

The implemented tool and the changing work practices resulted in the fulfilment of most of these requirements. With the implementation of the tool, a new information flow emerged, as described above: the system is the main source of tasks for maintainers now. Any tasks for them have to be entered into the system and maintenance work can no longer be requested informally by workers, through notes left on the desk, in quick conversations or via phone. Only in emergencies can requests be made via the phone, when a task must be addressed immediately. This has reduced the number of interruptions maintainers face It has improved clarity over what specifically needs to be done in each task, which in turn reduces time spent, for example by enabling maintainers to bring appropriate tools right away to the geographically distributed workplaces of the shopfloor. Perhaps most importantly it has reduced the number of tasks maintainers forget and has enabled them to create an overview of what needs to be done.

The tool has also increased transparency over the work of the maintainers and of the repair issues that need to be addressed. With the new system, foremen and management can perceive what maintainers are busy with and maintainers, therefore, receive recognition for their work. This transparency also leads to increased awareness by foremen over any potential issues that affect planning and production. As has been mentioned above, many workers are welders, who can be employed flexibly within the production line. The availability of a welder impacts production planning and thus a welder, who is unoccupied because a machine at a specific location broke down can be employed elsewhere, thereby reducing any losses in productivity. The tool and the process that leads to new tickets, which must be recorded and issued by foremen, increases this awareness over issues. Foremen now can also gain increased awareness about issues in upstream workplaces, which may result in changing machine setup and the order of production (Button and Sharrock, 1997). This therefore also addresses the previously identified challenge that maintainers and workers did not entirely agree on who is to report any issues. Maintainers assumed workers would report to foremen, foremen assumed maintainers would do so, which in practice then often led to maintenance issues being unreported, leaving foremen unaware. The tool and the process of registering tickets now ensure that foremen are aware of any issues, not just because they show up in the system, but also because they are the ones entering them there.

Both the speed and the depth of the appropriation also indicates that the intervention led to welcome changes. This is highlighted by three observations. Firstly, maintainers enforced the use of the tool and the new process it entailed. Whenever workers approached maintainers with a request for repair, they referred them to the tool and to their respective foreman to register the issue. This represents a significant change from the previous process, in which such informal approaches were in fact the main way of registering maintenance issues. Secondly, the tool was rolled out in the initial production line without the presence of the researchers and designers. The foremen introduced the tool to the production line managers during one of the shopfloor meetings and expressly told the researchers that their presence was not required. This was because there was already a significant level of familiarity with the tool, gained during the joint design process. The absence of the designers also did not result in any challenges—clearly, foremen were sufficiently familiar with the tool to explain its use to their colleagues and implement a reporting process. Thirdly, the tool was rolled out in other production lines and areas of the shopfloor, also without the involvement of the researchers and designers, and even without notifying them. This expansion was noticed when 34 new workplaces appeared as foremen created them while entering a new ticket. As was later discovered, this happened at the request of foremen from these other production lines who had learned about the app and wanted to use it within their own work contexts. It was then introduced and explained to them by the foremen of the initial production line, who also customized it accordingly.

The findings also show unexpected new practices relating to the entering of tickets as well as handling prioritization. In contrast to the pre-study, foremen and not workers can enter tickets into the system, because only former have the respective devices (tablets, desktop computers) at their disposal. However, when foremen are contacted by workers, both tend to discuss and enter the ticket cooperatively. This way, and due to the rejection of informal issue reports by maintenance, the intended flow of information changed to foremen being a central point of communication, rather than allowing workers to report directly in the new tool. Secondly, prioritization was handled differently than previously anticipated by all participants. Instead of shifting prioritization to higher management levels, our findings clearly show that initial estimations by those entering the tickets (mostly production line managers) mostly remain unchanged. Prioritization as represented in the tool, thus, may not serve as a crucial means for ordering maintainers work, but only serves as one aspect of situated decision making or is simply taken for granted. The fact that most priority changes are done during the meetings with maintainers and the head of production indicates that maintainers did not intervene into an explicit change of priorities within the new tool, but, however, do so indirectly by actually choosing their next tasks. This presents a shift from the initial intended process, such that maintainers have tools available to adapt the initial prioritization in a formal manner. These tools are seemingly ignored, and maintainers make small adjustments to prioritization ‘on the fly’, using the formally entered level of priority as orientation, but without any changes to priority as represented in the tool. Given the effort put into designing the formal prioritization process, this comes as a surprise. They even noted (during our second evaluation) that they open the app every day (or in between) to see if there are new issues reported, which also implies that the prioritization is not the principle factor in managing, planning, performing tasks.

**Figure 26.**
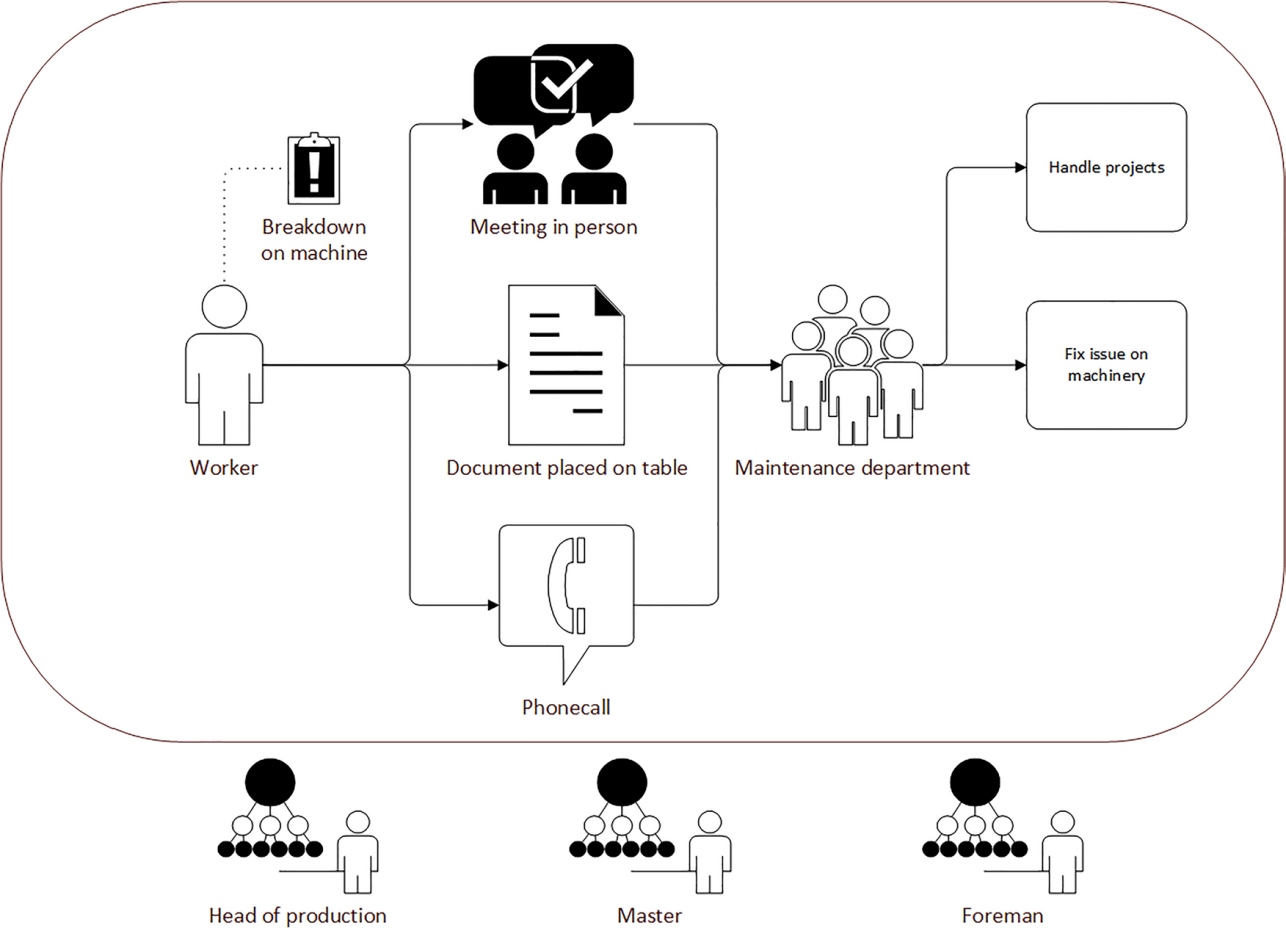
Simple presentation of the process before the project took place

Depicting the changes in the work practice on a process-level shows a much more structured workflow. Figure 26 shows a simple representation of the process we observed prior to our intervention. Although this process description is necessarily a simplification of the actual maintenance practice, it is noticeable that main parts of the organizational structure like Head of production, masters and foreman are outside the space where information is shared, and action takes place.

**Figure 27.**
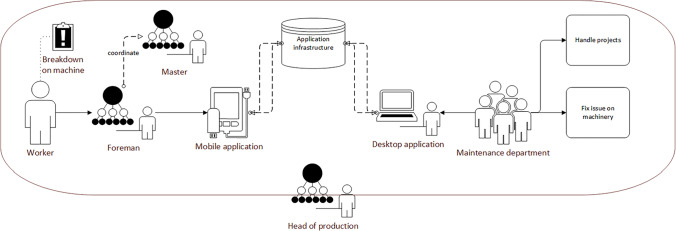
Simple presentation of the process after the project

Our presented actions throughout the study then resulted in an adapted process which is shown in Figure 27. The centralized application acts as a controlled medium over which the information about a breakdown is shared and maintainers can handle these as tickets as well as their long-term projects. Noticeable is also that foreman and masters are part of the space where information is shared basically through the fact that foreman have access to mobile devices to post a ticket to the maintenance department. Head of production is not fully integrated into this space as we have omitted some requirements like reports. These will be tackled in future developments and research. We are also aware of the fact that there are exceptions to this idealized process. There are for example occasions where information is still presented to the maintenance department according to the process in Fig. 26.

### Social nature of maintenance

Taken together, the findings of the pre-study and the evaluation of the intervention highlight an important characteristic, often overlooked in the study of maintenance: maintenance and repair activities are *socio*-technical activities rather than just technical, deterministic activities. This illustrated by various results from our design case study (Wulf et al., 2011, 2015).

Firstly, it becomes clear that maintenance and repair in this specific company is a highly cooperative activity, interdependent with many other activities taking place at the shopfloor. While officially the company divides different activities into ‘primary’ production activities and the ‘secondary’ activities which include maintenance, the activities are difficult to keep apart in practice. Primary activities depend on the coordination of tasks with maintenance, in case of acute machine failure, in which repair is necessary immediately for production to continue, but also future-oriented, preventive tasks, such as oiling a specific machine, which is not necessary at a specific moment to continue production, but within a specific timeframe to prevent damage or breakdown in the future. Such tasks often require the machine not to be in use, for which maintainers and operators need to coordinate a specific time. Maintainers depend on coordination with primary activities to carry out rather routine maintenance of infrastructure as well, such as changing lightbulbs, for which certain machines need to be not in use or need to be moved out of the way. Rather than being a separate activity, happening ‘on the side’ so to speak, in parallel with primary activities, maintenance work is deeply entangled with all other activities and in need of a high level of coordination.

Furthermore, a certain level of awareness about maintenance work is not only required by workers engaged in production activities, but also by foremen and other managerial roles, in order to plan. As mentioned already, if welders cannot do a specific task due to ongoing maintenance activities, these welders can potentially work on other tasks. For this, those responsible for planning activities need to be aware of any changes in the order of production, as Button and Sharrock (1997) have termed it, such as those caused by breakdown and repair activities. The new tool assists with this awareness, as it highlights which maintenance activities need to be taken care of and which ones are being taken care of either right now or in the near future, when maintainers move tickets within the system to a separate status board. Curiously, there also seemed to be a desire of foremen to be ‘in the know’, possibly for status or hierarchy reasons. This becomes apparent in the way the tool was finally rolled-out by the foremen. Initially, it was planned that workers would issue tickets in the system, similarly to the previous process in which they called maintainers directly or left notes on their desk. When the tool was introduced to the production line, it was decided that only foremen would be able to enter tickets, workers would need to approach them and enter the ticket together. While this was initially the case due to a shortage of devices and only planned for a shorter test phase, foremen later refused to change this, keeping in their central role as those issuing tickets.

Lastly, the process of prioritizing is also a highly coordinative activity, that requires specific knowledge and expertise on the side of the maintainers, as well as ‘organizational acumen’ (Tolmie and Rouncefield, 2016). Tasks are not selected by the maintainers according to the priority assigned to a task, but they translate the tickets into a paper-based to-do list of their own, for which they pick specific tickets, thereby re-assigning their own priority to each task. The pre-assigned priority of the ticket in the system is just one source of information for their own prioritization. Others are, for example, geographical location within the production site, such that tasks can be grouped together where they occur, especially if a smaller task can be taken care of while on route to the site of another task. Especially in early phases maintainers also reported having incentives to take care of smaller tasks quickly, to signal to workers that their requests in the system were taken seriously by them. Making this paper-based personal to-do list therefore requires the technical knowledge to assess how long each task might take, ‘organizational acumen’ to understand for example, which tasks might have priority for the different other positions that maintainers deal with (workers, foremen, managers, etc.), and how a re-prioritization might affect the ability of each of these persons to carry out their work. It also requires knowledge of the layout of the production site, to assess what can be done on route to other tasks.

Similar observations have been made by others, such as Orr (1996), Bobrow and Whalen (2002) or Yamauchi et al. (2003) in their studies of the practices of printer service technicians, who draw on a wide variety of sources and experimental practices to probe faulty printers, understand what the issue is and how it could be fixed, rather than following a manual. Similarly, Tolmie and Rouncefield (2016) report on the difficulty of creating actual organizational descriptions and ideals and the challenge of modelling of organizational processes. In our case, the tool, and the associated use patterns such as the issuing of tickets also embody specific organizational processes, but rather than fully prescribing them in a static manner they (necessarily) leave room to circumnavigate or adapt them as necessary in that specific moment. The described organizational developments are also the basis for projects on hands-on digitalization like predictive maintenance, technical advancements and incorporation of humans and machines. Organizational changes, like the awareness of different stakeholders in the process of maintenance, that maintenance in the future relies on data retrieved from products and processes, are a first step to prepare the basis for advanced technology (Daily and Peterson 2017; Sala et al. 2019). Selcuk (2017) stated that predictive maintenance ‘is a maintenance policy […] being practiced by the industry for many years’ which shows that even technological advancements in maintenance are subject to organizational decisions, structures, controls once again. Today’s capability of capturing lots of data spurs the implementation of concepts like predictive maintenance.

Interestingly, the introduction and appropriation of the tool reveals interesting insights into the dynamics of organizational hierarchies and accountabilities. These dynamics also ‘override’ design in a sense. While the tool was designed for all workers to report errors, in practice it is introduced and appropriated in such a way that the organizational hierarchy is strengthened, as only foremen issue tickets (and therefore set the initial priority of each task), affirming their position in the hierarchy. At the same time, however, the formal procedures of reporting and fixing errors, as represented in the tool, allow maintainers to take back control over their work. Even though foremen set initial priorities, maintainers are in control to set the final priority of each task and creating the order in which they are addressed. Their superiors’ priority is surely an important factor in determining order, but not the only one, as our study shows. Just like their foremen, however, they depend on the transparent overview over all outstanding maintenance tasks that the tool affords, in order to take control over the order of production. There is thus an interesting tension between design and appropriation with regards to organizational structure. This structure both leads to foremen ignoring design in favor of an affirmation of hierarchy, as well as to maintainers using the design to re-take control, yet also ignoring other prioritization affordances for the same purpose. Organizational commitment was also vital in terms of executing the project. The management in charge was committed to the project and initiated it. The attitude and commitment of maintainers in working on this project also made a huge difference and was a motivational factor. The enhanced interest of workers showed that the project was ‘on track’, resulting mainly from our participatory approach, and they came to see their engagement as ‘part of my job’ (Cooke, 2000). This study then also provides additional insights to existing studies on maintenance practices such as those by Betz (2010) or Lutters and Ackerman (2007), who focus on knowledge sharing and -management practices, such as repair history of a specific machine, rather than the coordinative and collaborative nature of maintenance work on an industrial shopfloor.

More importantly, however, this study provides a corrective to a large body of managerial literature in terms of how to understand and address maintenance. As we have mentioned earlier, this managerial literature largely focuses on strategies and best practices and on creating and implementing them (Wireman, 2004), drawing, for example, on perspectives such as ‘total cost of ownership’ (Gartner Group, 1997), and critically examined by Castellani et al. (2005). Such managerial perspectives and a focus on strategy necessarily represent abstract processes to be followed and require the quantification of the relevant parameters to overcome the absence of measures to assess maintenance, which has been lamented before (Raouf and Ben‐Daya, 1995). Our study shows that these models, again, cannot be simply applied into real-world maintenance practice, as they neglect subtle, hardly measurable interpersonal relationships and the means to maintain these e.g., avoiding displeasure among colleagues, re-affirming, or re-negotiating positions within the hierarchy, etc. This is not to say that managers are wrong (in general or in our particular case), but that a top-down perspective on maintenance as exhibited by this managerial literature is insufficient, as it leaves little room for the kind of situated decision-making we have witnessed here. An obvious example, mentioned above, is that, while access the ticketing system could be quantified when assessing the total cost of ownership, this would not account for the way other tasks are sometimes dealt with ‘on the way’ (see Castellani et al. (2005) for a similar problem). Curiously, as alluded to above, this discrepancy is also found in our case, as during the pre-study and subsequent workshops all participants, not only managers, expressed a preference for rigid prioritization procedures, which are in practice not followed through, even by the same people who requested them. Issues of maintenance then, in this case, are expressly not addressed by rigidly implementing specific procedures and treating maintenance as a purely technical activity to be optimized, but by leaving room within the tool for any necessary social processes that occur, and which cannot be prescribed, but are, as we pointed out above, situated, and contingent.

## Conclusion

In this paper we have presented findings from our study within a regional SME, focusing on processes around maintenance, maintainers workload, prioritization of issues and support of adjacent activities like reporting issues in production lines and negotiating about current problems. We critically discussed our findings collected through a Design Case Study (Wulf et al., 2011, 2015) comparing it with the current literature about strategies in maintenance and the view of maintenance as a technical secondary activity (see e.g. Wurhofer et al., 2018). Throughout our two-years-study, we engaged 13 individuals from the company from different hierarchical levels, with different perspectives on maintenance and with different roles in the process in interviews and workshops. Our findings show that maintenance is a highly entangled set of processes in the technical but also the organizational alignment of a company.

Qualitative studies of this kind are subject to some limitations. The set of participants in our study do not represent the majority of workers, maintainers or similar groups in SMEs and our organizational and technological intervention is only (for now) applicable for this specific case. Not least, SMEs in a domain such as this often work under severe cost and time constraints and maintenance activities are highly contingent on the type of machinery, its age, the level of technical know-wow required, and so on. Radical innovation in such circumstances can be problematic and it was clear in this particular context that it was dis-preferred. From our point of view, therefore, the important outcome in practical terms was the production of a working prototype that served an identifiable need and in academic terms, to assess the relationship between the design of a new technology of this kind and the way in which it is appropriated in practice. As we have seen in our evaluation our approach and the resulting technological artifact had a significant effect on the already established processes and resulted in an effective socio-technical outcome welcomed by all participants.

By taking the socio-technical nature of maintenance and its interdependent relations with other practices on the shopfloor into account, our intervention led to several changes in the practices around maintenance. These include an increased awareness amongst foremen and higher management of maintenance issues and the frequency and type of machine breakdowns, resulting in improved production planning, as well improved awareness of workload management issues on the side of maintainers. It assisted in the coordination of tasks between maintainers and other workers and enabled maintainers to react more appropriately to the issues at hand in a given moment, rather than reacting to ad hoc demands by workers.

Our study thereby makes two major contributions. Firstly, by providing a rich ethnographic account of maintenance practice in industrial contexts it adds to our understanding of such practices and highlights industrial maintenance as a promising field for CSCW research and interventions. Secondly, it provides an additional, alternative lens to a set of literature that focusses on managerial aspects of maintenance, trying to draw out and reflect on the positive effects of the development and implementation of strategies and best practices. Our study shows the situated and contingent nature of the coordinative aspects of maintenance work that exclude themselves from consideration in the more technical and managerialist literatures. This suggests that socio-technical interventions, such as ours, provide an improved sense of the relationship between orders of production and the contingencies which impact on them.

## Footnotes

1 Only men participated in our study.

2 Due to production schedules, vacations or sicknesses it was not possible to bring all participants together at any time in the project like for example during both of the workshops.

3 A Progressive Web App (PWA) is a state-of-the-art solution for cross platform mobile development. One of its core features is the offline capability, making it similar to native, local applications.

4 https://otrs.com/ and https://github.com/OTRS/otrs
